# Evaluating the Accuracy of Medical Information Generated by ChatGPT and Gemini and Its Alignment With International Clinical Guidelines From the Surviving Sepsis Campaign: Comparative Study

**DOI:** 10.2196/84251

**Published:** 2025-12-17

**Authors:** Dina Kutbi, Ehab Abou-Bakr, Hassan Mousa Haidar

**Affiliations:** 1 Department of Pharmacy King Fahad Armed Forces Hospital Jeddah Saudi Arabia; 2 Department of Business Jeddah International College Jeddah Saudi Arabia; 3 Department of Computer Science and Information Technology Jeddah International College Jeddah Saudi Arabia; 4 Department of Computer Engineering The Higher Institute of Engineering and Technology Elbehera Egypt

**Keywords:** sepsis, chatbots, artificial intelligence, AI, ChatGPT, medical information, Gemini, large language model

## Abstract

**Background:**

Assessment of medical information provided by artificial intelligence (AI) chatbots like ChatGPT and Google’s Gemini and comparison with international guidelines is a burgeoning area of research. These AI models are increasingly being considered for their potential to support clinical decision-making and patient education. However, their accuracy and reliability in delivering medical information that aligns with established guidelines remain under scrutiny.

**Objective:**

This study aims to assess the accuracy of medical information generated by ChatGPT and Gemini and its alignment with international guidelines for sepsis management.

**Methods:**

ChatGPT and Gemini were asked 18 questions about the Surviving Sepsis Campaign guidelines, and the responses were evaluated by 7 independent intensive care physicians. The responses generated were scored as follows: 3=correct, complete, and accurate; 2=correct but incomplete or inaccurate; and 1=incorrect. This scoring system was chosen to provide a clear and straightforward assessment of the accuracy and completeness of the responses. The Fleiss κ test was used to assess the agreement between evaluators, and the Mann-Whitney *U* test was used to test for the significance of differences between the correct responses generated by ChatGPT and Gemini.

**Results:**

ChatGPT provided 5 (28%) perfect responses, 12 (67%) nearly perfect responses, and 1 (5%) low-quality response, with substantial agreement among the evaluators (Fleiss κ=0.656). Gemini, on the other hand, provided 3 (17%) perfect responses, 14 (78%) nearly perfect responses, and 1 (5%) low-quality response, with moderate agreement among the evaluators (Fleiss κ=0.582). The Mann-Whitney *U* test revealed no statistically significant difference between the two platforms (*P*=.48).

**Conclusions:**

ChatGPT and Gemini both demonstrated potential for generating medical information. Despite their current limitations, both showed promise as complementary tools in patient education and clinical decision-making. The medical information generated by ChatGPT and Gemini still needs ongoing evaluation regarding its accuracy and alignment with international guidelines in different medical domains, particularly in the sepsis field.

## Introduction

In the medical field, precise diagnoses are essential to managing patients’ situations. When making clinical choices, clinicians often depend on their experience and a variety of case scenarios [1]. Sepsis and septic shock are two severe pathological conditions that are defined by the body’s reaction to an infection; they may result in organ failure and they have a high death rate. To increase the odds of survival, early diagnosis and prompt treatment are essential. Due to its vague symptoms and the wide range of patient reactions to infections, sepsis is difficult to detect [2]. Improved diagnostic assistance is thus required as the complexity of patients increases, especially those who require referrals to specialized departments like general internal medicine [1].

The assessment of artificial intelligence (AI)–driven models for providing medical information on sepsis and septic shock, as well as comparison of this information with international guidelines, is a burgeoning area of research. AI technologies have shown promise in enhancing diagnostic precision and therapeutic efficacy in sepsis management, yet their alignment with established guidelines like those of the Surviving Sepsis Campaign remains a critical area of evaluation [3]. AI models have been integrated into sepsis management for early detection, subtyping analysis, and prognosis assessment. These systems rely on high-granularity data from intensive care unit (ICU) settings to improve recognition and intervention capabilities, potentially leading to more precise treatment strategies [3]. The performance of these AI models can vary significantly depending on the complexity of the questions and the medical domain being addressed. For instance, ChatGPT’s accuracy is lower in responding to more complex medical science questions compared to simpler fracture prevention queries [4].

A previous study comparing ChatGPT (GPT-3.5) and Gemini found that the platforms had similar accuracy rates (71% and 70.5%, respectively) when answering microbiology questions [5]. However, their performance varied across different sections, with Gemini excelling in general microbiology and immunology, while ChatGPT performed better in applied microbiology [5]. In the context of local anesthesia for eye surgery, ChatGPT demonstrated slightly higher accuracy than Gemini (the scores were 4.71 and 4.61). However, traditional patient information leaflets outperformed both AI models in terms of accuracy and completeness [6]. For questions about immune-related adverse events, ChatGPT scored higher than Gemini in accuracy (3.87 vs 3.5) and completeness (3.83 vs 3.46), indicating generally reliable performance in this specific medical domain [7].

Even though several generative AI models have recently been built, ChatGPT remains one of the most popular [8]. Gemini is a user-friendly and efficient AI tool that has transformed ways to access and engage with various types of information by providing advanced, accurate, and relevant responses [9]. While AI platforms like ChatGPT and Gemini show promise in providing medical information, they are not without limitations. Their accuracy and reliability can vary, and they often lack the completeness and adherence to guidelines that traditional sources provide. For example, both ChatGPT and Gemini have been noted to produce factual inaccuracies, including fabricated citations and summaries, which raises concerns about their reliability as standalone sources of clinical information [10,11]. This study, therefore, aims to assess the accuracy of medical information generated by ChatGPT and Gemini and determine how they align with international guidelines for sepsis management.

## Methods

### Study Design

In this cross-sectional study, the 2021 Surviving Sepsis Guidelines [12] were used as the gold standard reference. The guidelines were rephrased as 18 questions (Multimedia Appendix 1), varying from simple, direct questions to questions about more complex clinical scenarios. The questions encompassed screening, diagnosis, management, and follow-up planning in order to cover all aspects of the guidelines.

Then, ChatGPT (GPT-4o) and Gemini 1.5 were prompted to answer the questions (Multimedia Appendices 2 and 3), and the responses generated by each of these large language models (LLMs) were evaluated for accuracy and alignment with the 2021 Surviving Sepsis Guidelines.

Seven independent intensive care physicians with 15 to 20 years of critical care experience were recruited for the evaluation. The recruited physicians blindly and independently scored the answers generated by ChatGPT and Gemini on a scale of 3 to 1 (3=correct, complete, and accurate; 2=correct but incomplete or inaccurate; 1=incorrect). For blinding, ChatGPT was assessed as “AI-Platform-1” while Gemini was assessed as “AI-Platform-2,” without naming the models.

### Data Analysis

The data in each group were analyzed with the Fleiss κ test to measure interrater agreement within the group. All responses were assessed independently by 7 physicians using a standardized 3-point scoring rubric (1 to 3). This rubric evaluated (1) scientific accuracy, (2) completeness of the response, and (3) correctness of the clinical reasoning. Responses scoring 3 points were categorized as perfect answers, those scoring 2 points as nearly perfect answers, and those scoring 1 point as low-quality answers. The percentages of perfect answers, nearly perfect answers, and low-quality answers were calculated for each platform. The Mann-Whitney *U* test was then used to assess any significant differences between the two platforms. Analysis used SPSS (version 18.0; IBM Inc) and differences were considered significant at a *P* value of less than .05.

### Ethical Considerations

Participating physicians gave their verbal approval and informed consent to participate. However, ethical approval does not apply to this study based on the laws and regulations of the Saudi National Committee of Bioethics guidelines (version 3; 2022).

## Results

The scores assigned by the 7 physicians to ChatGPT’s and Gemini’s responses to the 18 sepsis and septic shock questions are presented in Table 1 and Table 2, respectively. ChatGPT provided 5 (28%) perfect responses to questions about sepsis management, which were fully aligned with guidelines and recommendations. These included questions about source control, nutrition and glycemic control, antibiotic coverage for patients at high risk of multidrug resistance, antimicrobial stewardship in patients with an unconfirmed infectious diagnosis, and antibiotic treatment in patients with community-acquired pneumonia (CAP) regardless of procalcitonin (PCT) level. ChatGPT provided nearly perfect responses to 12 (67%) of the 18 questions; these responses had issues that included missing the time frame for ICU admission, the antibiotics administration time (within the golden hour), or the dosage for corticosteroids; recommending an inappropriate route of administration for neuromuscular blocking agents; not specifying Ringer lactate as the preferred resuscitation fluid of choice; and not mentioning ventilation measures or information about other screening and assessment tools. However, ChatGPT responded to a question about venous thromboembolism (VTE) prophylaxis with a low-quality answer that contradicted the guideline and included incorrect information about the renal adjustment dose of enoxaparin.

Among its 18 responses, Gemini only provided 3 (17%) that were perfect and fully aligned with guideline recommendations about source control and antimicrobial stewardship in patients with an unconfirmed infectious diagnosis and provided comprehensive information about quick sequential organ failure assessment and other assessment tools. Among the 18 responses, Gemini provided 14 nearly perfect answers (78%; Table 3); these answers were missing information on dosage, admission, drug timing, ventilator measures, and nutrition timing approaches. However, in response to a question on antibiotic coverage in patients with a high risk of multidrug resistance, while Gemini’s answer included information that aligned with the guideline recommendations, it also suggested irrelevant and incorrect alternative regimen examples, and it also provided 1 overall misleading answer about antibiotic treatment for CAP according to PCT level (Table 2).

The Fleiss κ values for both AI models highlighted the differences in interrater agreement levels and showed that ChatGPT had a score of 0.656, indicating a substantial agreement between the evaluators. However, Gemini had a Fleiss κ score of 0.582, indicating moderate agreement between the evaluators. The Mann-Whitney *U* test returned a *U* statistic of 184 and a *P* value of .48, indicating that there was no statistically significant difference between the average scores of the two AI models.

**Table 1:** Scores for ChatGPT.

Questions	Evaluator								Average score		Comments
	1	2	3	4	5	6	7				
Q1	3	2	3	3	2	3	3	2.7		Missed information—no time frame (within 6 hours).	
Q2	2	2	2	2	2	2	2	2		Inaccurate answer according to guideline recommendations.	
Q3	2	2	2	2	2	2	2	2		Missed information—no time frame (within 1 hour).	
Q4	3	3	3	3	3	3	3	3		None.	
Q5	2	2	2	2	2	2	2	2		Inaccurate answer according to guideline recommendations.	
Q6	2	2	2	2	2	2	2	2		Inaccurate answer as the doses were missed.	
Q7	3	2	3	3	2	3	3	2.7		Inaccurate answer according to guideline recommendations, but the correct guideline was highlighted in the answer.	
Q8	3	3	3	3	2	3	3	2.8		Inaccurate answer according to guideline recommendations.	
Q9	3	3	3	3	3	3	3	3		None.	
Q10	2	2	3	3	3	2	3	2.6		Inaccurate answer according to guideline recommendations.	
Q11	3	2	3	3	2	2	3	2.6		Missed information—posthospital rehabilitation program.	
Q12	3	3	3	3	3	3	3	3		None.	
Q13	3	2	3	3	2	3	3	2.7		Missed information—some measures and treatment duration (no plateau measures or prone time mentioned).	
Q14	2	2	2	2	2	2	2	2		Inaccurate answer according to guideline recommendations.	
Q15	3	3	3	3	3	3	3	3		None.	
Q16	3	3	3	3	3	3	3	3		None.	
Q17	2	2	2	2	2	2	2	2		Inaccurate answer according to guideline recommendations.	
Q18	2	2	2	2	2	2	1	1.8		Inaccurate answer according to guideline recommendations. Also, the dose mentioned was not accurate.	

**Table 2:** Scores for Gemini.

Question	Evaluator								Average score		Comments
	1	2	3	4	5	6	7				
Q1	3	2	3	3	2	3	3	2.7		Missed information—no time frame (admission within 6 hours).	
Q2	2	2	2	2	2	2	2	2		Inaccurate answer according to guideline recommendations.	
Q3	2	2	2	2	2	2	2	2		Missed information—no time frame (within 1 hour).	
Q4	3	3	3	3	3	3	3	3		None.	
Q5	3	2	3	3	2	2	3	2.6		Missed information—no doses mentioned.	
Q6	2	2	2	2	2	2	2	2		Missed information—no doses or time frame mentioned.	
Q7	3	2	3	3	2	2	2	2.4		Inaccurate answer according to guideline recommendations.	
Q8	2	2	2	2	2	2	2	2		Inaccurate answer according to guideline recommendations.	
Q9	3	2	3	3	3	3	3	2.8		Inaccurate answer according to guideline recommendations.	
Q10	3	2	3	3	3	2	3	2.7		Inaccurate answer according to guideline recommendations.	
Q11	3	2	3	3	2	3	3	2.7		Missed information—post-hospital rehabilitation program.	
Q12	3	2	3	3	3	3	1	2.6		Answer is fully aligned with guideline recommendation but contains one incorrect choice of alternative drug regimen mentioned in the explanation (piperacillin/tazobactam is not used for organisms resistant to meropenem, while ceftazidime/avibactam is used).	
Q13	2	2	3	3	3	3	3	2.7		Missed information—duration of prone position was not mentioned. Also, the preference of high PEEP^a^ over low PEEP.	
Q14	3	3	3	3	3	3	3	3		None.	
Q15	3	3	3	3	3	3	3	3		None.	
Q16	1	1	2	1	1	1	1	1.1		Overall answer was not aligned with guideline recommendation (PCT^b^ is not the goal measure for deescalation of antibiotics and CAP^c^ treatment should be started regardless of PCT level).	
Q17	2	2	2	2	2	2	2	2		Inaccurate answer according to guideline recommendations.	
Q18	2	2	2	2	2	2	2	2		Inaccurate answer according to guideline recommendations and missed information (no doses mentioned).	

^a^PEEP: positive end-expiratory pressure.

^b^PCT: procalcitonin.

^c^CAP: community-acquired pneumonia.

**Table 3:** Percentages of perfect, nearly perfect, and low-quality answers to questions (n=18).

Scores	ChatGPT, n (%)	Gemini, n (%)
3 (perfect)	5 (28)	3 (17)
2 to <3 (nearly perfect)	12 (67)	14 (78)
1 to <2 (low quality)	1 (5)	1 (5)

## Discussion

This study evaluated the responses generated by ChatGPT and Gemini to 18 questions on septic shock and sepsis and compared the responses with guideline recommendations. Although ChatGPT responded perfectly to 5 questions and Gemini to 3 questions, some responses were inadequate and most were inaccurate. They could not provide accurate information on the timeframe for ICU admission or the administration of antibiotics, and most responses were missing information on the dosage of certain vasoactive drugs, ventilation measures, neuromuscular blocking agents, and the best route of administration according to the guidelines, suggesting that their medical knowledge is limited. These results corroborate the observations of Cascella et al [13], who reported that although these AI technologies may facilitate the acceleration of therapeutic interventions and patient education, there is a chance that they could provide incorrect suggestions.

AI has seen huge advancements in recent years, leading to impressive progress in a variety of forms, including AI chatbots. Because AI chatbots such as ChatGPT and Gemini can respond to almost any inquiry with thorough and human-like replies, they have drawn a large user base in a variety of industries. According to reports, ChatGPT is used for many medical purposes, including medical teaching, writing, and recording. It has recently been announced that ChatGPT can pass the United States Medical Licensing Examination, which is considered the gold standard, indicating that it may have important medical uses [14].

However, the growing volume and accessibility of medical data pose significant difficulties for physicians. A growing number of physicians and patients are using chatbots to help make medical information easier to understand and more accessible. It is thus critical to assess the accuracy and dependability of chatbot responses, particularly as patients are increasingly depending on these responses to guide their medical decisions [15]. Serious pathological conditions such as sepsis and septic shock are defined by the body’s reaction to an infection, which may result in organ failure and a high death rate. To increase the odds of survival, early diagnosis and prompt treatment are essential. Due to its vague symptoms and the wide range of patient reactions to infections, sepsis is difficult to diagnose [2]. According to several previous studies, initial iterations of chatbots provided information that was easy to understand and somewhat accurate. Still, they also gave inadequate, erroneous, or outdated responses [16]. For example, ChatGPT was shown to be generally accurate in answering broad inquiries regarding osteoporosis; however, the replies to queries based on the National Osteoporosis Criteria Group criteria were only 61.3% correct [17].

There are subtle differences between ChatGPT and Gemini when it comes to generating clinical information. Due to its deep learning capabilities, Gemini may perform very well in specialized fields like ophthalmology [18]. However, for wider applications, ChatGPT’s adaptability and accessibility make it very useful for general patient education in a variety of medical fields. Its capacity to produce understandable and customized content is especially appreciated in patient education, providing a more dynamic and captivating approach than conventional techniques and some AI alternatives [19]. This observation aligns with the results of this study, which showed that ChatGPT outperformed Gemini in its recommendations for the choice of antibiotics for an ICU patient diagnosed with septic shock suspected of being due to gram-negative infection and for an ICU patient with possible CAP. In contrast, Gemini provided a complete response and alternate assessment tools that differed from the quick sequential organ failure assessment score for diagnosing sepsis.

Furthermore, when asked for recommendations on VTE prophylaxis in critically ill patients, apart from the fact that both AI chatbots failed to recommend proper prophylaxis according to the guidelines, which discourage the use of mechanical VTE plus pharmacological prophylaxis in favor of pharmacological prophylaxis alone, ChatGPT added incorrect information on the renal adjustment dose of enoxaparin (30 mg subcutaneously twice a day instead of 30 mg once daily). These differences between the two AI chatbots might be due to differences in their training databases, design patterns, application algorithms, and updates [20].

The statistical analysis using Fleiss κ indicated substantial agreement for ChatGPT and moderate agreement for Gemini in the quality of their responses, reflecting their relative consistency in adhering to clinical guidelines. Additionally, the Mann-Whitney *U* test supported the absence of a statistically significant difference in their performance. These findings align with previous research that directly compared ChatGPT and Gemini in delivering effective medical information, demonstrating no significant statistical difference between the two platforms [21]. This underlines the comparable potential of both general-purpose and specialized AI systems in supporting clinical decision-making, provided their outputs are critically evaluated and supplemented with human oversight.

Although this study provides a robust set of evidence on the accuracy of ChatGPT and Gemini in generating medical information, its limitations need to be considered. It primarily examined the accuracy and alignment of generalist and commonly used LLM-generated medical information with international clinical guidelines, and it did not include medically fine-tuned and domain-specific LLMs, such as Med-PaLM and BioGPT, which might perform differently on domain-specific tasks.

In conclusion, despite current limitations, AI chatbots like ChatGPT and Gemini show promise as complementary tools in patient education and clinical decision-making, potentially enhancing health care delivery by providing quick access to medical information [22]. The medical information generated by ChatGPT and Gemini still needs continuous evaluation for accuracy, reliability, and alignment with international guidelines in different medical domains. Thus, their use for medical information and treatment should only occur after consultation with a human health care professional. Finally, there is still a need for improvement and further research through training of the AI chatbots using evidence-based sepsis management datasets, including clinical guidelines (such as the Surviving Sepsis Campaign) and real-world case studies.

## Appendix

Multimedia Appendix 1 2021 Surviving Sepsis Guidelines, rephrased as 18 questions.

Multimedia Appendix 2 Responses to questions by ChatGPT.

Multimedia Appendix 3 Responses to questions by Gemini.

## Abbreviations

AI: artificial intelligence
CAP: community-acquired pneumonia
LLM: large language model
PCT: procalcitonin
VTE: venous thromboembolism

## Data Availability

All data generated or analyzed during this study are included in this published article and its supplementary information files.
