# Supplementary material for: Evaluating the Accuracy of Medical Information Generated by ChatGPT and Gemini and Its Alignment With International Clinical Guidelines From the Surviving Sepsis Campaign: Comparative Study
Source: JMIR Form Res. 2025 Dec 17;9:e84251. doi: 10.2196/84251 (PMC12756662; doi:10.2196/84251)
Supplement: Multimedia Appendix 1 [file formative_v9i1e84251_app1.pdf]

Q1 What are effective strategies for hospitals to enhance the early detection and treatment of sepsis and septic shock in a timely manner?

For hospitals and health systems, we recommend using a performance improvement program for sepsis, including sepsis screening for acutely ill, high-risk patients and standard operating procedures for treatment. For adults with sepsis or septic shock who require ICU admission, we suggest admitting the patients to the ICU within 6 hours.

Q2 What is the initial recommended crystalloid fluid of choice for sepsis resuscitation? Mention the full strategy

Sepsis and septic shock are medical emergencies, and we recommend that treatment and resuscitation begin immediately. For patients with sepsis-induced hypoperfusion or septic shock, we suggest that at least 30 mL/kg of intravenous (IV) crystalloid fluid should be given within the first 3 h of resuscitation. We suggest using dynamic measures to guide fluid resuscitation over physical examination or static parameters alone. We suggest measuring blood lactate. We suggest using capillary refill time to guide resuscitation as an adjunct to other measures of perfusion. We recommend an initial target mean arterial pressure (MAP) of 65 mm Hg over higher MAP targets. Also, we suggest the use of Albumin in patients who have received large amounts of crystalloids.

Q3 What is the approach to start antimicrobial therapy when sepsis is recognized?

We recommend administering antimicrobials immediately, ideally within 1 h of recognition. We recommend rapid assessment of the likelihood of infectious versus non-infectious causes of acute illness. Whenever possible, this should be completed within 3 h of presentation so that a decision can be made as to the likelihood of an infectious cause of the patient’s presentation and timely antimicrobial therapy provided if the likelihood of sepsis is thought to be high. We suggest a time-limited course of rapid investigation, and if concern for infection persists, the administration of antimicrobials within 3 h from the time when sepsis was first recognized.

Q4 What is the approach to address source control in sepsis and septic shock?

We recommend rapidly identifying or excluding a specific anatomical diagnosis of infection that requires emergent source control and implementing any required source control intervention as soon as medically and logistically practical. We recommend prompt removal of intravascular access devices that are a possible source of sepsis or septic shock after other vascular access has been established.

Q5 What are the recommended strategies for managing vasoactive drugs in septic shock?

For adults with septic shock, we recommend using norepinephrine as the first-line agent over other vasopressors. In settings where norepinephrine is not available, epinephrine or dopamine can be used as an alternative, but we encourage efforts to improve the availability of norepinephrine. Special attention should be given to patients at risk for arrhythmias when using dopamine and epinephrine. For adults with septic shock on norepinephrine with inadequate MAP levels, we suggest adding vasopressin instead of escalating the dose of norepinephrine. In our practice, vasopressin is usually started when the dose of norepinephrine is in the range of 0.25–0.5 µg/kg/min. For adults with septic shock and inadequate MAP levels despite norepinephrine and vasopressin, we suggest adding epinephrine. For adults with septic shock, we suggest against using terlipressin.

For adults with septic shock and cardiac dysfunction with persistent hypoperfusion despite adequate volume status and arterial blood pressure, we suggest either adding dobutamine to norepinephrine or using epinephrine alone. For adults with septic shock and cardiac dysfunction with persistent hypoperfusion despite adequate volume status and arterial blood pressure, we suggest against using levosimendan.

Q6 Under which circumstances should corticosteroids be used in septic shock?

For adults with septic shock and an ongoing requirement for vasopressor therapy we suggest using IV corticosteroids Weak recommendation; moderate quality of evidence Remark The typical corticosteroid used in adults with septic shock is IV hydrocortisone at a dose of 200 mg/day given as 50 mg intravenously every 6 h or as a continuous infusion. It is suggested that this is commenced at a dose of norepinephrine or epinephrine ≥ 0.25 mcg/kg/min at least 4 h after initiation.

Q7 What is the approach of using neuromuscular blocking agents in adults' sepsis induced ARDS?

For adults with sepsis induced moderate-severe ARDS, we suggest using intermittent NMBA boluses, over NMBA continuous infusion.

Q8 What are the recommendations regarding the routine use Bicarbonate therapy and IV vitamin C in sepsis and septic shock?

- For adults with septic shock and hypoperfusion-induced lactic acidemia, we suggest against using sodium bicarbonate therapy to improve hemodynamics or to reduce vasopressor requirements. For adults with septic shock, severe metabolic acidemia (pH ≤ 7.2) and AKI (AKIN score 2 or 3), we suggest using sodium bicarbonate therapy.
- The guideline suggests against the use of IV Vitamin C in sepsis or septic shock

Q9 What are the approaches for nutrition and glycemic control in patient with sepsis and septic shock?

- For adult patients with sepsis or septic shock who can be fed enterally, we suggest early (within 72 h) initiation of enteral nutrition.
- For adults with sepsis or septic shock, we recommend initiating insulin therapy at a glucose level of ≥ 180 mg/dL (10 mmol/L) Following initiation of an insulin therapy, a typical target blood glucose range is 144–180 mg/dL (8–10 mmol/L).

Q10 (Complex) 65-year-old man who presented with symptoms of sepsis. He was admitted with high fever, tachycardia, low blood pressure, and confusion. Blood cultures are pending, but his clinical presentation strongly suggests septic shock, requiring immediate resuscitation. The attending physician is briefing the team on the optimal management strategy. The discussion focuses on the choice of intravenous fluid for resuscitation to restore his hemodynamic status. As the team explores different fluid options including crystalloids, albumin, starches and synthetic colloids, such as gelatin. What will be the best choice for him?

- We recommend using crystalloids as first-line fluid for resuscitation.
- We suggest using balanced crystalloids instead of normal saline for resuscitation.
- We suggest using albumin in patients who received large volumes of crystalloids over using crystalloids alone.

- We recommend against using starches and gelatin for resuscitation.

Q11 (Complex) 58-year-old woman, was admitted to the ICU with severe sepsis due to a urinary tract infection. She required mechanical ventilation for five days and spent ten days in the ICU. After a successful treatment, she is now stable and ready for discharge from the hospital. What is your plan for follow up?

For adults with sepsis or septic shock and their families, we recommend the clinical team provide the opportunity to participate in shared decision making in post-ICU and hospital discharge planning to ensure discharge plans are acceptable and feasible. We suggest using a critical care transition program, compared to usual care, upon transfer to the floor. We recommend reconciling medications at both ICU and hospital discharge. For adult survivors of sepsis and septic shock and their families, we recommend including information about the ICU stay, sepsis and related diagnoses, treatments, and common impairments after sepsis in the written and verbal hospital discharge summary. We recommend assessment and follow-up for physical, cognitive, and emotional problems after hospital discharge. We suggest referral to a post-critical illness follow-up program if available. For adult survivors of sepsis or septic shock receiving mechanical ventilation for > 48 h or an ICU stay of > 72 h, we suggest referral to a post-hospital rehabilitation program.

Q12 (Complex) 72-year-old male diagnosed with septic shock suspected to be due to gram-negative infection with risk factors of recent hospitalization, prior antibiotic exposure, and a past history of urinary tract infections resistant to first-line antibiotics. ICU doctors started him on meropenem and ciprofloxacin plus vancomycin. What are your recommendations regarding antibiotics choices?

- For adults with sepsis or septic shock and high risk for multidrug resistant (MDR) organisms, we suggest using two antimicrobials with gram-negative coverage for empiric treatment over one gram-negative agent
- For adults with sepsis or septic shock and low risk for MDR organisms, we suggest against using two Gram-negative agents for empiric treatment, as compared to one Gram-negative agent

-For adults with sepsis or septic shock, we suggest against using double gram-negative coverage once the causative pathogen and the susceptibilities are known

Q13 (Complex) 58-year-old female patient in the ICU. She was recently diagnosed with severe acute respiratory distress syndrome (ARDS) as a complication of sepsis secondary to community-acquired pneumonia. She is on mechanical ventilation, and you are part of the multidisciplinary team responsible for managing her ventilatory settings to optimize her respiratory function while minimizing further lung injury. What are the key elements of ventilatory management that can prevent ventilator-induced lung injury?

In accordance with current guidelines, for adults with sepsis-induced moderate-severe ARDS, we recommend using prone ventilation for greater than 12 hours daily. This intervention has been shown to improve oxygenation and reduce mortality in these patients. Use low tidal volume ventilation (6 mL/kg of predicted body weight) and maintain plateau pressures ≤ 30 cm H2O for ARDS management. For adults with sepsis-induced severe ARDS, we recommend using an upper limit goal for plateau pressures of 30 cm H2O. This limit is preferred over higher plateau pressures to prevent ventilator-induced lung

injury and to protect the lungs from barotrauma, thus improving the patient's outcomes. Also, for moderate to severe ARDS we suggest to use higher PEEP over lower PEEP.

Q14 (Complex) 60-year-old female, type 2 diabetes, arrives at the hospital with complaints of severe fatigue, shortness of breath, and a productive cough. In the emergency department, her vital signs are as follows: temperature 38.3°C (100.9°F), heart rate 105 beats per minute, respiratory rate 24 breaths per minute, and blood pressure 118/70 mmHg. Her oxygen saturation is 92% on room air. The medical team considers assessing her for possible sepsis. The quick Sequential Organ Failure Assessment (qSOFA) score, based on altered mentation, low blood pressure, and increased respiratory rate, is considered as part of the initial screening process. What might be the limitations of relying solely on the qSOFA score for identifying sepsis? Give a rational compared to other assessment tools

We recommend against using qSOFA compared to SIRS, NEWS, or MEWS as a single screening tool for sepsis or septic shock.

Rationale: The qSOFA uses 3 variables to predict death and prolonged ICU stay in patients with known or suspected sepsis: a Glasgow Coma Score < 15, a respiratory rate ≥ 22 breaths/min and a systolic blood pressure ≤ 100 mmHg. When any two of these variables are present simultaneously the patient is considered to be qSOFA positive.

Studies have shown that qSOFA is more specific but less sensitive than having two of four SIRS criteria for early identification of infection-induced organ dysfunction. Neither SIRS nor qSOFA are ideal screening tools for sepsis.

Similar findings have also been found when comparing against the National Early warning Score (NEWS) and the Modified Early warning Score (MEWS) [44]. Although the presence of a positive qSOFA should alert the clinician to the possibility of sepsis in all resource settings; given the poor sensitivity of the qSOFA, the panel issued a strong recommendation against its use as a single screening tool.

Q15 (Complex) 52-year-old gentleman with a history of hypertension, presents to the emergency department with high fever, chills, and confusion. On physical examination, he appears acutely ill with a temperature of 38.9°C (102°F), a heart rate of 112 beats per minute, and a blood pressure of 86/58 mmHg. Initial blood tests reveal elevated white blood cell count and elevated lactate levels, suggesting possible sepsis. However, blood and urine cultures are negative after 48 hours, and no clear source of infection is identified. The medical team suspects sepsis or septic shock and initiates empiric broadspectrum antibiotics. Over the next 24 hours, his condition stabilizes, but no evidence of bacterial infection is found from subsequent tests and imaging. Considering the recommendation on empiric antimicrobials, what should the healthcare team consider doing next in managing his treatment?

For adults with suspected sepsis or septic shock but unconfirmed infection, we recommend continuously reevaluating and searching for an alternative diagnosis and discontinuing empiric antimicrobials if an alternative cause of illness is demonstrated or strongly suspected.

Q16 (Complex) A 64-year-old woman is admitted to the medical ICU with possible community-acquired pneumonia. The patient is initiated on ceftriaxone and azithromycin. Chest radiography reveals focal infiltrate. On admission, she is dyspneic with a respiratory rate of 33 breaths/minute. Her vital signs and laboratory values are as follows: blood pressure 90/50 mm Hg, heart rate 101 beats/minute, WBC

18 x 103 cells/mm3, and lactate 4.2 mmol/L. A PCT is obtained on admission. The results are available 12 hours after antibiotics are initiated. The PCT result is 0.1 mcg/L. What is your action regarding antibiotics?

For adults with suspected sepsis or septic shock, we suggest against using procalcitonin plus clinical evaluation to decide when to start antimicrobials, as compared to clinical evaluation alone. Published guidelines for the management of community-acquired pneumonia recommend initiation of antimicrobials for patients with community-acquired pneumonia regardless of procalcitonin level.

Q17 (Complex) 78-year-old male patient with medical history of diabetes mellitus, hypertension, previous abdominal surgery, and antibiotic exposure. Presented to the hospital with fever, chills, abdominal pain, tachycardia, hypotension, and confusion. Initially diagnosed with signs of septic shock, started fluid resuscitation and antimicrobials, including meropenem. What is your recommendation regarding this regimen?

For adults with sepsis or septic shock at high risk of methicillin resist- ant staph aureus (MRSA), we recommend using empiric antimicrobials with MRSA coverage over using antimicrobials without MRSA coverage. We suggest the use of empirical anti-fungal therapy in adult patients with sepsis or septic shock who are at risk of fungal infection.

Q18 67-year-old male, known case of Hypertension, type 2 Diabetes, Chronic Obstructive Pulmonary Disease (COPD). Admitted to the ICU 48 hours ago following a severe case of community-acquired pneumonia resulting in respiratory failure. Currently intubated and mechanically ventilated, sedated, and on vasopressors to maintain blood pressure. Current Medications: Vasopressors (Norepinephrine), Sedation (Propofol), Antibiotics (Ceftriaxone and Azithromycin), Insulin sliding scale for glucose management, Vital Signs: Blood Pressure 100/60 mmHg on vasopressors, Heart Rate 85 bpm, Respiratory Rate 18 bpm (via ventilator). Lab Results: Elevated inflammatory markers, creatinine is stable at 1.1 mg/dL. What are the possible approaches regarding VTE (Venous Thromboembolism) prophylaxis?

For adults with sepsis or septic shock, we recommend using pharmacological VTE prophylaxis unless a contraindication to such therapy exists. We recommend using low molecular weight heparin (LMWH) over unfractionated heparin (UFH) for VTE prophylaxis. We suggest against using mechanical VTE prophylaxis in addition to pharmacological prophylaxis, over pharmacologic prophylaxis alone.
