# Supplementary material for: Evaluating the Accuracy of Medical Information Generated by ChatGPT and Gemini and Its Alignment With International Clinical Guidelines From the Surviving Sepsis Campaign: Comparative Study
Source: JMIR Form Res. 2025 Dec 17;9:e84251. doi: 10.2196/84251 (PMC12756662; doi:10.2196/84251)
Supplement: Multimedia Appendix 2 [file formative_v9i1e84251_app2.pdf]

Q1 What are effective strategies for hospitals to enhance the early detection and treatment of sepsis and septic shock in a timely manner?

Enhancing early detection and treatment of sepsis and septic shock in hospitals involves a combination of clinical protocols, training, and technology integration. Here are several effective strategies:

1- **Standardized Protocols:** Implement evidence-based protocols for sepsis recognition and management, such as the Surviving Sepsis Campaign guidelines. These protocols should include early and broad-spectrum antibiotic administration, timely fluid resuscitation, and the use of metrics to track patient outcomes.

2- **Rapid Screening Tools:** Develop and utilize standardized screening tools for early identification of sepsis. Tools like the qSOFA (Quick Sequential Organ Failure Assessment) can aid in quickly assessing at-risk patients.

3- **Education and Training:** Regular training sessions for healthcare staff about the signs and symptoms of sepsis and the importance of early intervention. Simulation training can be effective in practice and readiness.

4- **Interdisciplinary Teams:** Establish multidisciplinary teams that include physicians, nurses, pharmacists, and infection control specialists to manage sepsis cases. Implementing rapid response teams can facilitate quicker interventions.

5- **Monitoring and Alerts:** Use electronic health record (EHR) systems equipped with alerts for clinicians when certain criteria indicative of sepsis is met. This can include abnormal vital signs or lab results.

6- **Sepsis Protocol Activation:** Create a clear protocol for rapid sepsis response activation that triggers a set course of action when sepsis is suspected, ensuring timely treatment.

7- **Performance Metrics:** Monitor and analyze sepsis-related outcomes and adherence to protocol to ensure continuous improvement. This can help identify areas needing attention and improve overall care.

8- **Patient and Family Education:** Engage patients and families in understanding the signs of sepsis, which can lead to quicker reporting of symptoms and faster initiation of care.

9- **Quality Improvement Initiatives:** Regularly assess and refine sepsis protocols through quality improvement initiatives, including feedback loops from the staff involved in care delivery.

By combining these strategies, hospitals can improve their timelines for detecting and treating sepsis, ultimately enhancing patient outcomes.

Score:

Comments:

Q2 What is the initial recommended crystalloid fluid of choice for sepsis resuscitation? Mention the full strategy

The initial recommended crystalloid fluid of choice for sepsis resuscitation is normal saline (0.9% sodium chloride) or lactated Ringer's solution. Both are commonly used in clinical settings. Here's a full strategy for sepsis resuscitation based on the Surviving Sepsis Campaign guidelines:

Initial Resuscitation Strategy for Sepsis and Septic Shock:

Initial Assessment and Recognition:

Quickly assess the patient for signs of sepsis using screening tools (e.g., qSOFA).

Obtain a thorough history and assess vital signs (heart rate, blood pressure, respiratory rate, temperature).

Fluid Resuscitation: Initiate intravenous fluid resuscitation as soon as sepsis is suspected.

Administer a minimum of 30 mL/kg of crystalloid fluid (normal saline or lactated Ringer's) within the first 3 hours. This may be adjusted based on the patient's response.

Monitoring Response: Regularly monitor the patient's response to fluid resuscitation:

Vital signs (blood pressure, heart rate), urine output, mental status, reassess after the initial fluid bolus, and consider further fluids based on clinical response.

Vasopressor Support: If hypotension persists despite adequate fluid resuscitation, initiate vasopressor therapy to maintain a mean arterial pressure (MAP) of at least 65 mmHg. Norepinephrine is the first-choice vasopressor. Other options, such as epinephrine can be considered if additional support is needed.

Antibiotic Therapy: Administer broad-spectrum intravenous antibiotics within the first hour of recognizing sepsis. Choose empiric therapy based on local guidelines and suspected sources of infection.

Source Control: Identify and address the underlying source of infection as soon as possible. This may include drainage of abscesses, removal of infected devices, or surgical intervention.

Monitoring and Reassessment: Continuously monitor vital signs, laboratory markers, and patient's overall status. Adjust treatment based on ongoing assessment. Use biomarkers (e.g., lactate levels) to help guide resuscitation goals and further management.

Considerations for Additional Therapies: Consider the role of adjunctive therapies like corticosteroids if septic shock persists despite adequate fluid and vasopressor therapy. Evaluate the use of monitoring techniques for advanced hemodynamics if the patient's condition is critical.

Following these steps can help improve the outcomes for patients with sepsis and septic shock by ensuring timely and effective resuscitation. Always adapt the approach based on individual patient needs and clinical judgment.

Score:

Comments:

Q3 What is the approach to start antimicrobial therapy when sepsis is recognized?

When sepsis is recognized, initiating antimicrobial therapy promptly is crucial for improving patient outcomes. Here's an approach to starting antimicrobial therapy in suspected sepsis:

Approach to Initiate Antimicrobial Therapy in Sepsis:

Early Recognition: Identify signs and symptoms of sepsis, including fever, chills, tachycardia, hypotension, altered mental status, and signs of infection.

Obtain Cultures (Before Antibiotics): Consider obtaining blood cultures and other relevant cultures (e.g., urine, sputum, wound) before starting antibiotics. This helps identify the causative organism and guide therapy. However, do not delay antibiotic administration for culture collection.

Administer Antimicrobials: Timing: Administer broad-spectrum intravenous (IV) antibiotics as soon as possible, ideally within the first hour of recognizing sepsis.

Choice of Agents: Select broad-spectrum antibiotics based on: The most common organisms associated with the suspected source of infection (e.g., community-acquired pneumonia, urinary tract infection, intra-abdominal infection). Local microbiological resistance patterns. Patient history, allergies, and prior antibiotic use.

Example: For community-acquired pneumonia: Consider options like ceftriaxone plus azithromycin or moxifloxacin. **For urinary tract infections:** Options might include piperacillin-tazobactam, cefepime, or meropenem, depending on severity and local resistance patterns. **For intra-abdominal infections:** Consider piperacillin-tazobactam or meropenem. **For healthcare-associated infections:** Broader coverage may be needed, including antipseudomonal agents.

De-Escalation: Once culture results are available and the specific pathogen is identified, adjust the antimicrobial therapy to narrow-spectrum agents that are effective against the identified organisms. Base de-escalation decisions on culture results, clinical response, and patient history.

Duration of Therapy: Initially, continue antibiotics for 7 to 10 days, depending on the source and severity of the infection, and reassess the need for continued therapy based on clinical improvement.

Reassessment: Continuously monitor the patient's clinical status and response to therapy. Adjust treatment based on effectiveness, culture results, and clinical guidelines.

Documentation: Ensure proper documentation of the patient's symptoms, culture results, antibiotic initiation time, and any changes to the treatment plan.

By following this structured approach, healthcare providers can optimize the management of sepsis and enhance the chances of a favorable outcome for the patient.

Score:

Comments

Q4 What is the approach to address source control in sepsis and septic shock?

Addressing source control in sepsis and septic shock is a critical component of effective management. Source control refers to the identification and elimination of the underlying infection or source of sepsis. Here’s an approach to effectively address source control:

Approach to Source Control in Sepsis and Septic Shock:

Early Identification: Assess for potential sources of infection based on patient history, physical examination, and laboratory findings. Common sources include pneumonia, urinary tract infections, abdominal infections, skin and soft tissue infections, and devices (e.g., catheters).

Rapid Imaging: Utilize imaging studies (e.g., ultrasound, CT scans, X-rays) to localize the infection or to assess for abscesses, fluid collections, or other complications.

Microbiological Cultures: Obtain microbiological cultures (e.g., blood, urine, sputum, wound swabs) before any definitive surgical or invasive procedures when possible. This aids in identifying pathogens and guides targeted antibiotic therapy.

Surgical Intervention: Consider surgical intervention for drainage of abscesses, debridement of infected tissue, removal of infected devices, or any surgical procedures necessary to control the source of infection. If bacterial infections are associated with an intra-abdominal cause (e.g., perforated bowel), definitive surgical management is crucial.

Drainage of Fluid Collections: For conditions like abdominal sepsis or empyema, perform percutaneous or surgical drainage of infected fluid collections (e.g., abscesses, pleural effusions).

Device Removal: If an infection is associated with intravascular devices (e.g., central lines), remove the infected devices promptly to control the source of infection.

Assessment for Additional Sources: Continuously reassess the patient for other potential sources of infection throughout treatment. Persistent infections may require further interventions.

Monitor Clinical Response: Monitor the patient’s clinical response to therapy and source control interventions. Improvement typically involves stabilizing vitals, improving laboratory markers (e.g., lactate levels), and resolving symptoms.

Coordination of Care: Collaborate with surgical teams, infectious disease specialists, and other relevant healthcare providers to ensure comprehensive evaluation and management.

Documentation: Document findings, interventions, and patient responses in detail to maintain clear communication among all members of the healthcare team.

By addressing source control methodically, healthcare providers can significantly improve treatment outcomes in patients with sepsis and septic shock, ultimately preventing further complications and supporting recovery.

Scor:

Comments:

Q5 What are the recommended strategies for managing vasoactive drugs (epinephrine, norepinephrine, vasopressin, dopamine, dobutamine, terlipressin and levosimendan) in septic shock?

Managing vasoactive drugs in septic shock is essential for improving hemodynamic stability and ensuring adequate perfusion of vital organs. Here's an overview of recommended strategies for the use of various vasoactive agents in septic shock:

Recommended Strategies for Managing Vasoactive Drugs in Septic Shock:

Norepinephrine: First-line Agent: Norepinephrine is the first-line vasopressor for septic shock due to its effective action in increasing mean arterial pressure (MAP) and improving organ perfusion. **Dosing:** Start at 0.05 to 0.5 mcg/kg/min and titrate based on hemodynamic response, typically aiming for a MAP of at least 65 mmHg. **Administration:** Use a central line if the dosage exceeds 0.1 mcg/kg/min to minimize the risk of extravasation and tissue necrosis.

Epinephrine: Second-line Agent: Epinephrine can be used if additional support is needed or if there is significant cardiogenic shock. **Dosing:** Initiate at 0.01 to 0.5 mcg/kg/min, titrate according to clinical response. **Caution:** It has more pronounced chronotropic and inotropic effects compared to norepinephrine, which may lead to increased heart rate and myocardial oxygen demand.

Vasopressin: Adjunctive Therapy: Vasopressin can be added to norepinephrine when adequate blood pressure is not achieved with norepinephrine alone. **Dosing:** Start at 0.03 units/min, and adjust if needed. May help reduce norepinephrine requirements. **Use:** Consider in patients with severe septic shock or those requiring high doses of norepinephrine.

Dopamine: Limited Use: Dopamine is generally not recommended for routine use in septic shock due to its variable effects and increased risk of adverse outcomes. **Indications:** It may be used in patients with bradycardia or those who also have significant myocardial dysfunction. **Dosing:** Start at 2 to 20 mcg/kg/min, but consider alternative agents for the overall management of septic shock.

Dobutamine: Inotropic Support: Dobutamine can be used in cases of septic shock where myocardial contractility is critically compromised. **Dosing:** Start at 2 to 20 mcg/kg/min, titrate based on cardiac output and systemic vascular resistance. **Monitoring:** Continuous monitoring is essential for assessing cardiac function and hemodynamic response.

Terlipressin: Alternative Vasopressor: Terlipressin may be considered for patients with vasodilatory shock and a low systemic vascular resistance. **Dosing:** Initiate at 0.5 to 2 mg every 4 to 6 hours, titrate as needed based on hemodynamic response. **Use:** More commonly used in certain regions, particularly in acute cirrhosis or hepatorenal syndrome.

Levosimendan: Inotropic and Vasodilatory Effects: Levosimendan provides both inotropic support and vasodilation, making it useful in cases of septic shock with reduced cardiac output. **Dosing:** Start with a loading dose of 6 to 12 mcg/kg over 10 minutes, followed by a continuous infusion of 0.1 to 0.4 mcg/kg/min. **Monitoring:** Carefully monitor for hypotension and other hemodynamic changes.

General Considerations:

Monitoring: Continuous hemodynamic monitoring is crucial to evaluate the effectiveness of therapies and adjust dosages accordingly.

Individualization: Tailor therapy based on the patient's hemodynamic status, underlying conditions, and response to treatment.

Combination Therapy: Using a combination of vasoactive agents may be necessary, depending on the severity of shock and the hemodynamic response.

Wean Off Therapy: Gradually wean off vasoactive drugs as the patient stabilizes, while monitoring for signs of persistent shock or instability.

By carefully managing these vasoactive medications, healthcare providers can optimize hemodynamic support for patients in septic shock, improving outcomes and facilitating recovery.

Score:

Comments:

Q6 Under which circumstances should corticosteroids be used in septic shock?

Corticosteroids can play a role in the management of septic shock, particularly in certain circumstances. Here are the key considerations regarding their use:

Circumstances for Corticosteroid Use in Septic Shock:

Adrenal Insufficiency: Patients with known or suspected primary adrenal insufficiency (e.g., Addison's disease) should receive corticosteroid therapy, as they may not be able to mount an adequate adrenal response to stress.

Severe Septic Shock: Corticosteroids may be considered in patients with severe septic shock who exhibit refractory hypotension despite adequate fluid resuscitation and vasopressor therapy.

Moderate to High-Dose Vasopressor Requirement: The use of corticosteroids is recommended for patients requiring moderate to high doses of vasopressors (e.g., norepinephrine) to achieve adequate mean arterial pressure (MAP) (usually ≥65 mmHg).

Clinical Guidelines and Recommendations: The Surviving Sepsis Campaign guidelines recommend administering hydrocortisone (e.g., 200 mg/day in continuous or intermittent doses) for patients with septic shock who do not respond to fluid resuscitation and vasopressor therapy.

Time of Initiation: If corticosteroids are to be used, they should be administered as early as possible in the course of septic shock for potential benefits.

Improved Outcomes: Some evidence suggests that corticosteroids can improve outcomes in septic shock, including organ dysfunction, duration of vasopressor therapy, and overall mortality, particularly in the context of severe sepsis or septic shock.

Important Considerations:

Contraindications: Use corticosteroids cautiously or avoid them in some patients, such as those with certain infections where steroids may exacerbate the condition or delay the immune response.

Tapering: If steroids are started in the context of septic shock, it may not be necessary to taper doses when the underlying infection is treated and the patient stabilizes.

Adverse Effects: Be aware of potential side effects of corticosteroids, including hyperglycemia and increased risk of secondary infections.

Monitoring: Regularly reassess the patient's hemodynamic status and organ function while on corticosteroid therapy, and be prepared to adjust treatment based on their clinical response.

By using corticosteroids appropriately in septic shock, healthcare providers can help manage hemodynamics and support recovery, particularly in patients with severe or refractory cases.

Score:

Comments:

Q7 What is the approach of using neuromuscular blocking agents in adults' sepsis-induced ARDS?

The use of neuromuscular blocking agents (NMBAs) in adults with sepsis-induced acute respiratory distress syndrome (ARDS) requires careful consideration and adherence to clinical guidelines. Here's an approach to the use of NMBAs in this context:

Approach to Using Neuromuscular Blocking Agents in Sepsis-Induced ARDS:

Indication for Use: NMBAs may be considered in patients with moderate to severe ARDS to improve mechanical ventilation outcomes, especially in cases where the patient is experiencing substantial ventilator-induced lung injury due to high tidal volumes or pressures.

Recommended Agents: Commonly used NMBAs include cisatracurium and vecuronium. Cisatracurium is often preferred due to its metabolism via non-specific plasma esterases and tissue esterases, making it safer in critically ill patients.

Timing: Consider the early initiation of NMBAs within the first 48 hours of intubation in patients with severe ARDS, particularly if they are experiencing significant patient-ventilator desynchrony.

Dosing: Administer NMBAs using a continuous infusion approach, typically starting with recommended dosing based on clinical practice guidelines. For example, cisatracurium may be infused at 1-2 mcg/kg/min.

Monitoring: Continuously monitor and assess the patient's level of sedation and paralysis. Use a peripheral nerve stimulator to guide NMBA dosing and minimize over-paralysis. Ensure that patients are adequately sedated to prevent awareness during paralysis.

Duration of Therapy: Utilize NMBAs for the shortest duration necessary, typically up to 48 hours. Evaluate the need for ongoing therapy based on clinical improvement and wean off when appropriate.

Sedation: Prior to the use of NMBAs, ensure appropriate sedation and analgesia are provided to minimize discomfort. Use sedatives like propofol or dexmedetomidine in conjunction with NMBAs.

Weaning Off: Gradually wean patients off NMBAs based on clinical status. Monitor for neuromuscular recovery frequently, and assess readiness for extubation once the underlying ARDS improves.

Potential Benefits: Clinical studies have shown that the use of NMBAs can help improve oxygenation and reduce the duration of mechanical ventilation in patients with moderate to severe ARDS.

Assess Risks: Be aware of potential complications associated with NMBA use, including prolonged neuromuscular blockade, muscle weakness, and critical illness myopathy.

Clinical Guidelines:

Follow institutional protocols and guidelines, such as the Surviving Sepsis Campaign or ARDS-specific studies (e.g., the ACURASYS trial), for the use of NMBAs. These guidelines recommend their use in specific contexts and emphasize the importance of monitoring and safety.

By applying this approach, healthcare providers can effectively manage sedation and improve mechanical ventilation strategies in patients suffering from sepsis-induced ARDS while minimizing risks and adverse effects.

Score:

Comments:

Q8 What are the recommendations regarding the routine use of Bicarbonate therapy and IV vitamin C in sepsis and septic shock?

The recommendations regarding the routine use of bicarbonate therapy and intravenous (IV) vitamin C in sepsis and septic shock are based on current clinical evidence and guidelines. Here's a summary of the recommendations for both therapies:

Bicarbonate Therapy: Indications: Routine bicarbonate therapy is not recommended for the treatment of metabolic acidosis in septic shock unless the patient has a pH < 7.15. The use of sodium bicarbonate

can have potential risks, such as fluid overload and hypernatremia, without significantly improving outcomes in most patients.

Guidelines: The Surviving Sepsis Campaign guidelines recommend against the routine use of bicarbonate for patients with severe metabolic acidosis unless necessary to correct a low pH.

Consideration of bicarbonate therapy should be individualized, based on the patient's clinical status and metabolic acidosis severity.

Monitoring: If bicarbonate therapy is administered, it should be closely monitored for effects on acid-base balance and electrolyte levels to avoid complications.

Intravenous Vitamin C (Ascorbic Acid):

Current Evidence: The evidence regarding the routine use of intravenous vitamin C in the treatment of sepsis and septic shock is mixed and still under investigation. Some observational studies and small clinical trials suggested that high-dose vitamin C may have a beneficial role in reducing organ dysfunction and improving outcomes in patients with sepsis.

Recommendations:

As of now, the routine use of IV vitamin C is not recommended for all patients with sepsis or septic shock due to insufficient high-quality evidence to support its efficacy. However, in specific contexts, particularly in trials, high-dose IV vitamin C might be considered as an adjunctive treatment under clinical supervision.

Ongoing Research: Several larger randomized controlled trials are ongoing to determine the safety and efficacy of high-dose vitamin C for patients with sepsis, which may provide clearer guidance in the future.

Combination Therapy: Some studies have considered the use of vitamin C in combination with other therapies (e.g., corticosteroids or thiamine), leading to a potential synergistic effect. However, definitive conclusions regarding combination therapy are not established.

Conclusion:

Currently, the routine use of bicarbonate therapy and IV vitamin C in sepsis and septic shock is not supported by strong evidence. Bicarbonate may be necessary only in severe cases of acidosis with low pH, while vitamin C may be considered cautiously within clinical studies. Healthcare providers should focus on evidence-based practices and guidelines, ensuring that any adjunct therapies are used judiciously and monitored closely for effectiveness and safety.

Score:

Comments:

Q9 (simple/direct-management) What are the approaches for nutrition and glycemic control in patient with sepsis and septic shock?

Nutrition and glycemic control are critical components of the management of patients with sepsis and septic shock. Here are the recommended approaches for both:

Nutrition Approaches:

Early Enteral Nutrition (EEN): Initiate enteral nutrition (EN) as soon as hemodynamic stability allows, typically within 24 to 48 hours of admission to the intensive care unit (ICU). Early enteral feeding has been associated with improved outcomes, including reduced infection rates and shorter hospital stays.

Individualized Nutritional Assessment: Assess the patient's nutritional needs based on factors such as weight, age, sex, and severity of illness. Use predictive equations (e.g., the Harris-Benedict equation or the Mifflin-St Jeor equation) to estimate calorie needs. Consider using the Indirect Calorimetry method if available for a more precise measurement of energy expenditure.

Target Caloric Intake: Aim for approximately 20-30 kcal/kg/day based on the patient's needs and tolerance. Adjust this target as the patient's condition changes. Provide protein supplementation to meet the protein needs, typically around 1.2 to 2.0 g/kg/day, depending on the severity of illness and catabolism.

Gastrointestinal Function Monitoring: Monitor for feeding tolerance and gastrointestinal function, adjusting the feeding regimen as necessary (e.g., if the patient develops abdominal distension or intolerance). Regularly assess the patient's weight and nutritional status to guide ongoing nutrition management.

Use of Parenteral Nutrition (if necessary): If enteral nutrition is not feasible or well-tolerated after 7-10 days, consider initiating parenteral nutrition (PN) to ensure adequate caloric delivery. However, parenteral nutrition should not be started until enteral feeding is maximized or intolerance persists.

Glycemic Control Approaches:

Target Blood Glucose Levels: The recommended target for blood glucose levels in critically ill patients is generally between 140-180 mg/dL (7.8-10.0 mmol/L). Avoid tight glycemic control (<140 mg/dL) as it may lead to increased risk of hypoglycemia and has not shown additional benefits in critically ill patients.

Insulin Therapy: Use intravenous insulin to manage hyperglycemia in patients with sepsis and septic shock who are unable to maintain target glucose levels with diet alone. Start insulin infusion if blood glucose exceeds 180 mg/dL, titrating doses to achieve target glucose levels.

Frequent Monitoring: Monitor blood glucose levels frequently (e.g., every 1-2 hours) initially, then adjust the frequency based on stability and response to treatment. Blood glucose levels should be monitored during enteral or parenteral nutrition initiation or changes in therapy.

Address Underlying Causes: Identify and manage any underlying causes of hyperglycemia (e.g., stress response, infection, inadequate insulin production) as part of a comprehensive approach to patient care.

Team-based Approach: Collaborate with a multidisciplinary team, including dietitians and endocrinologists, to optimize nutrition and glycemic control plans tailored to the individual patient's needs.

By implementing these evidence-based approaches to nutrition and glycemic control, healthcare providers can support the recovery of patients with sepsis and septic shock, improve metabolic stability, and enhance overall outcomes.

Score:

Comments:

Q10 65-year-old man who presented with symptoms of sepsis. He was admitted with high fever, tachycardia, low blood pressure, and confusion. Blood cultures are pending, but his clinical presentation strongly suggests septic shock, requiring immediate resuscitation. The attending physician is briefing the team on the optimal management strategy. The discussion focuses on the choice of intravenous fluid for resuscitation to restore his hemodynamic status. As the team explores different fluid options, including crystalloids, albumin, starches, and synthetic colloids, such as gelatin. What will be the best choice for him?

In a 65-year-old man presenting with symptoms of septic shock requiring immediate resuscitation, the best choice for intravenous fluid for resuscitation is crystalloids, specifically normal saline (0.9% sodium chloride) or lactated Ringer's solution.

Rationale:

Evidence-Based Guidelines: The Surviving Sepsis Campaign guidelines recommend crystalloids as the first-line fluid for the resuscitation of patients with sepsis and septic shock. Crystalloids are preferred due to their efficacy, safety profile, and cost-effectiveness.

Fluid Composition: Normal saline (0.9% sodium chloride) provides isotonic fluid replacement and helps expand intravascular volume. Lactated Ringer's solution, which contains electrolytes and lactate, can also be beneficial, particularly in managing acidosis.

Avoidance of Synthetic Colloids: There is evidence suggesting that synthetic colloids (e.g., hydroxyethyl starch, gelatin) are associated with a higher risk of nephrotoxicity and do not provide additional benefits over crystalloids in septic shock management.

Albumin Considerations: Although albumin can be used in specific situations (e.g., for patients with significant hypoproteinemia or when fluid resuscitation is challenging), it is typically not the first choice for initial resuscitation in septic shock. Crystalloids are generally adequate for most patients unless there is an indication for albumin use.

Conclusion:

Given the guidelines and clinical evidence, crystalloids should be the preferred choice for initial fluid resuscitation in this patient with septic shock. The team should initiate treatment with isotonic crystalloids while monitoring the patient's hemodynamic response, fluid balance, and any potential complications.

Score:

Comments:

Q11 A 58-year-old woman was admitted to the ICU with severe sepsis due to a urinary tract infection. She required mechanical ventilation for five days and spent ten days in the ICU. After a successful treatment, she is now stable and ready for discharge from the hospital. What is your plan for follow-up?

For a 58-year-old woman who has been successfully treated for severe sepsis due to a urinary tract infection and is now stable and ready for discharge, a comprehensive follow-up plan is essential to address her health needs and ensure a smooth recovery. Here's a structured follow-up plan:

Follow-Up Plan After Discharge:

Scheduled Follow-Up Appointments: Arrange follow-up appointments with her primary care physician (PCP) or infectious disease specialist within 1-2 weeks post-discharge to reassess her health status and recovery. If necessary, schedule visits with specialists (e.g., urologist) if there are underlying issues related to her urinary tract infection.

Monitoring for Complications: Educate the patient on the signs and symptoms of potential complications to watch for after discharge, such as: Fever, Increased malaise or fatigue, Changes in urinary symptoms (e.g., dysuria, urgency, frequency), Shortness of breath or difficulty breathing. Encourage the patient to seek medical attention promptly if any concerning symptoms arise.

Assessment of Functional Status: Evaluate her overall recovery, including assessment of functional status, mobility, and ability to perform activities of daily living (ADLs) during follow-up appointments. Refer to rehabilitation services (physical therapy, occupational therapy) if she exhibits weakness or functional impairment resulting from her ICU stay.

Nutritional Assessment: Ensure that the patient receives appropriate nutritional counseling to support recovery, especially if she has experienced weight loss, muscle weakness, or difficulty with appetite during her illness.

Psychosocial Support: Assess for any psychological effects related to her ICU stay (e.g., anxiety, depression, post-traumatic stress disorder) and provide appropriate referrals for mental health support if needed. Consider social work consultations to assist with any necessary resources or support systems.

Continued Medication Management: Review and manage any ongoing medications initiated during her hospital stay. Ensure that she understands her discharge medications, including any antibiotics if she requires an extended course. Discuss medication adherence and potential side effects.

Vaccination Review: Ensure that her immunizations are up to date, especially for influenza and pneumococcal vaccines, to reduce the risk of future infections.

Education for Patient and Family: Provide education on self-care practices, including: Recognition of infection symptoms. Proper hygiene and preventive measures for urinary tract infections involve family or caregivers in discharge planning and education for better support at home.

Coordination of Care: Ensure continuity of care by coordinating communication between all healthcare providers involved in her care, including outpatient physicians and specialists.

By implementing this follow-up plan, the patient will have access to the necessary support and resources while transitioning out of hospital care, ultimately enhancing her recovery and reducing the risk of readmission.

Score:

Comments:

Q12 72-year-old male diagnosed with septic shock suspected to be due to gram-negative infection with risk factors of Recent hospitalization, prior antibiotic exposure, and a past history of urinary tract infections resistant to first-line antibiotics. ICU doctors started him on meropenem and ciprofloxacin plus vancomycin. What are your recommendations regarding antibiotic choices?

In managing a 72-year-old male diagnosed with septic shock suspected to be due to a gram-negative infection, particularly in the presence of risk factors such as recent hospitalization, prior antibiotic exposure, and a history of urinary tract infections (UTIs) resistant to first-line antibiotics, careful consideration of antibiotic selection is essential. Here are recommendations regarding antibiotic choices for this patient:

Recommendations for Antibiotic Choices:

Initial Empiric Therapy: Broad-Spectrum Coverage: The current regimen of meropenem (a carbapenem effective against many gram-negative bacteria, including extended-spectrum beta-lactamase-producing organisms) is appropriate given the patient's risk factors and history.

Ciprofloxacin: Including ciprofloxacin provides additional coverage for resistant gram-negative organisms, especially Pseudomonas aeruginosa, and may also cover Enterobacteriaceae.

Vancomycin: The addition of vancomycin is warranted since it provides coverage against potential methicillin-resistant Staphylococcus aureus (MRSA), especially in cases of severe sepsis or septic shock with skin or soft tissue infection sources.

Review and De-Escalation: After initial cultures (e.g., blood, urine) are obtained, and sensitivities are available, it's crucial to de-escalate therapy based on the identified pathogens and their resistance profiles. Avoid unnecessary broad-spectrum therapy to reduce the risk of antibiotic resistance and adverse effects.

Adjustment Based on Culture Results:

Once the microbiological lab results are available: If a specific resistant organism (e.g., a resistant gram-negative) is identified, switch to a more targeted antibiotic that is effective against the isolated pathogen. Consider consulting with infectious disease specialists to optimize therapy based on resistance patterns and individual patient factors.

Duration of Therapy: The duration of antibiotic therapy should be guided by clinical response, source control, and microbiological findings. Typically, for sepsis, a duration of 7 to 10 days is often recommended, but this may vary based on the source of sepsis and individual patient circumstances.

Renal Considerations: Given the patient's age and potential for renal impairment (common in elderly patients), monitor renal function closely. Dosage adjustments may be necessary, especially for meropenem and ciprofloxacin.

Monitoring for Toxicity and Side Effects: Monitor for any adverse effects of the antibiotics, particularly for nephrotoxicity and neurotoxicity from carbapenems and the risk of QT prolongation with ciprofloxacin.

Summary: The current antibiotic regimen of meropenem, ciprofloxacin, and vancomycin provides broad-spectrum coverage appropriate for a patient with suspected septic shock and significant risk factors.

Ongoing assessment, culture results, and sensitivity patterns should guide de-escalation and adjustments to ensure effective and safe antibiotic therapy tailored to the patient's needs. Consulting an infectious disease specialist may provide additional support for complex cases.

Score:

Comments:

Q13 (Complex/scenario-management) 58-year-old female patient in the ICU. She was recently diagnosed with severe acute respiratory distress syndrome (ARDS) as a complication of sepsis secondary to community-acquired pneumonia. She is on mechanical ventilation, and you are part of the multidisciplinary team responsible for managing her ventilatory settings to optimize her respiratory function while minimizing further lung injury. What are the key elements of ventilatory management that can prevent ventilator-induced lung injury? Mention the full strategy

Managing a patient with severe acute respiratory distress syndrome (ARDS) requires careful consideration of ventilatory settings to optimize oxygenation and ventilation while minimizing the risk of ventilator-induced lung injury (VILI). Here's a comprehensive strategy for ventilatory management in this context: Key Elements of Ventilatory Management to Prevent Ventilator-Induced Lung Injury (VILI):

Use of Low Tidal Volumes: Target Tidal Volume: Set tidal volumes between 4-6 mL/kg of predicted body weight (PBW) to minimize overdistension of alveoli and reduce barotrauma. Predicted Body Weight Calculation: Use appropriate equations to calculate PBW based on height and gender.

Maintain Adequate Positive End-Expiratory Pressure (PEEP): PEEP Settings: Utilize moderate levels of PEEP (usually between 5-15 cm H_2O) to help recruit collapsed alveoli, improve oxygenation, and prevent atelectasis. Individualized PEEP: Adjust PEEP according to the patient's lung mechanics and oxygenation response. Monitor dynamic compliance and perform PEEP titration to find the optimal level.

Choose Appropriate Modes of Ventilation: Assist-Control Ventilation (AC) or Pressure-Control Ventilation (PC): These modes facilitate patient-ventilator synchrony and allow for safer ventilation strategies. Consider Volume-Assist/Control: Ensure that the delivered volume does not exceed set limits to prevent overdistention in volume control modes.

Oxygenation and Permissive Hypercapnia: Target SpO_2 Levels: Aim for oxygen saturation levels around 88-95% (PaO_2 55-80 mmHg) to avoid excessive oxygen toxicity. Accept Elevated CO_2 Levels: Use permissible hypercapnia as part of lung-protective strategies, accepting higher levels of carbon dioxide (pH 7.20-7.30) to avoid lung injury while maintaining volume protection.

Careful Monitoring and Adjustment of Ventilatory Settings: Frequent Reassessment: Regularly monitor arterial blood gases (ABGs), oxygenation, and lung mechanics to guide adjustments in ventilation strategy. Respiratory System Compliance: Regularly assess compliance to detect changes in lung function and adjust ventilatory parameters accordingly.

Prone Positioning: Consider Prone Positioning: If feasible, implement prone positioning in patients with severe ARDS, as it has been shown to improve oxygenation and reduce mortality risk.

Maintain Safety: Ensure safe and proper management when transitioning to the prone position with attention to securing equipment and monitoring for potential complications.

Sedation and Analgesia: Adequate Sedation: Provide appropriate sedation to enhance patient comfort and support ventilation efforts while avoiding agitation that may lead to barotrauma or polytrauma.

Use Neuromuscular Blockade: In cases of severe ARDS with significant patient-ventilator desynchrony, consider using neuromuscular blockers for a short period to facilitate compliance.

Avoid Excessive Respiratory Rates: Control Respiratory Rate: Set respiratory rates judiciously to avoid dynamic hyperinflation and auto-PEEP, balancing adequate ventilation with minimizing airway pressures.

Preventing Complications: Monitoring for VILI Signs: Watch for signs of barotrauma or polytrauma, which may necessitate reevaluating ventilator settings and further management.

Team Approach: Collaborate with the multidisciplinary team, including respiratory therapists, nurses, and physicians, to optimize care throughout the respiratory management process.

Summary

The overall strategy for managing a patient with ARDS includes implementing a lung-protective ventilation strategy by using low tidal volumes, appropriate PEEP, and modes of ventilation tailored to the patient's needs. Regular monitoring and adjustments based on clinical status and lung mechanics are vital to minimizing VILI while promoting optimal respiratory function.

Score:

Comments:

Q14 (Complex/scenario-screening/diagnosis) 60-year-old female, type 2 diabetes, arrives at the hospital with complaints of severe fatigue, shortness of breath, and a productive cough. In the emergency department, her vital signs are as follows: temperature 38.3°C (100.9°F), heart rate 105 beats per minute, respiratory rate 24 breaths per minute, and blood pressure 118/70 mmHg. Her oxygen saturation is 92% on room air. The medical team considers assessing her for possible sepsis. The quick Sequential Organ Failure Assessment (qSOFA) score, based on altered mentation, low blood pressure, and increased respiratory rate, is considered as part of the initial screening process. What might be the limitations of relying solely on the qSOFA score for identifying sepsis? Give a rational compared to other assessment tools

R solely on the Quick Sequential Organ Failure Assessment (qSOFA) score for identifying sepsis has limitations, as it may not fully capture the complex nature of sepsis. Here are some key limitations compared to other assessment tools:

Limitations of qSOFA:

Sensitivity Issues: The qSOFA score has been found to have moderate sensitivity but lower specificity. This means it may not identify all patients with sepsis, potentially missing cases, especially in initial presentations where vital signs may not be severely altered.

Narrow Focus on Three Parameters: qSOFA considers only three criteria: altered mentation, respiratory rate ≥ 22 breaths per minute, and systolic blood pressure ≤ 100 mmHg. This limited approach may

overlook other crucial indicators of severe infection or organ dysfunction, such as urinary output, lactate levels, or temperature changes.

Overlooked Clinical Context: The score does not account for certain populations, such as elderly patients, those with chronic illnesses, or patients on medications affecting vital signs (e.g., betablockers). These factors can alter heart rate or blood pressure readings and skew qSOFA results.

Inability to Capture Dynamics: qSOFA provides a snapshot in time but does not reflect changes in a patient's condition over time, which is critical in sepsis management. A patient may initially score low but develop significant deterioration shortly thereafter.

Comparison with Other Assessment Tools:

SOFA Score: The full Sequential Organ Failure Assessment (SOFA) score offers a more comprehensive evaluation of organ function and includes multiple systems (cardiovascular, respiratory, hepatic, coagulation, renal, and neurologic). It allows for a more detailed assessment of the severity of organ dysfunction, which is especially important in critically ill patients. The SOFA score is particularly useful for tracking changes in organ function over time, which can be critical in managing sepsis.

SIRS Criteria: Systemic Inflammatory Response Syndrome (SIRS) criteria include additional parameters like temperature and white blood cell count. Although less specific for sepsis, SIRS criteria can help identify systemic infection in a broader range of susceptible patients. It can be used in conjunction with qSOFA or SOFA for a more robust assessment.

Clinical Judgment: Assessing the patient's overall clinical picture, including their presenting symptoms and laboratory and imaging results, is essential for diagnosing sepsis. Clinical judgment based on history, signs, and expert evaluation should always complement scoring systems.

Lactate Measurement: Measuring serum lactate levels can provide vital information about tissue hypoperfusion and aid in identifying septic shock. Lactate levels greater than 2 mmol/L are associated with poor outcomes in sepsis, making lactate measurement a valuable addition to any assessment strategy.

Conclusion: While the qSOFA score is a useful tool for rapid screening of sepsis, it should not be the sole assessment method used for diagnosis. Its limitations in sensitivity and the narrow focus of its criteria necessitate the incorporation of complementary assessment tools like the SOFA score, clinical judgment, and laboratory findings to ensure a comprehensive evaluation of the patient's condition and assist in prompt and effective management of sepsis.

Score:

Comments:

Q15 52-year-old gentleman with a history of hypertension presents to the emergency department with high fever, chills, and confusion. On physical examination, he appears acutely ill with a temperature of 38.9°C (102°F), a heart rate of 112 beats per minute, and a blood pressure of 86/58 mmHg. Initial blood tests reveal an elevated white blood cell count and elevated lactate levels, suggesting possible sepsis. However, blood and urine cultures are negative after 48 hours, and no clear source of infection is identified. The medical team suspects sepsis or septic shock and initiates empiric broad-spectrum antibiotics. Over the next 24 hours, his condition stabilizes, but no evidence of bacterial infection is found from subsequent tests and imaging. Considering the recommendation on empiric antimicrobials, what should the healthcare team consider doing next in managing his treatment?

In managing a patient like the 52-year-old gentleman who initially presented with signs of sepsis or septic shock but has since stabilized with negative blood and urine cultures, the healthcare team should consider the following steps:

1. **Reassess the Need for Ongoing Antibiotics:** Review the necessity of continuing broad-spectrum antibiotics since no clear source of infection has been identified, and cultures remain negative. **Recommendation:** If the patient's clinical condition has stabilized and there is no evidence of ongoing infection, consider de-escalating or discontinuing antibiotics to minimize the risk of antibiotic resistance, adverse drug reactions, and Clostridium difficile infection.

2. **Evaluate for Other Causes:** Investigate alternative diagnoses that may explain the acute illness. Potential considerations might include: Non-bacterial infections: Consider viral infections (e.g., influenza, COVID-19), fungal infections, or atypical bacteria that may not be easily identified in routine cultures. Non-infectious causes: Assess for non-infectious conditions that can mimic sepsis, such as malignancy, pancreatitis, or autoimmune disorders.

3. **Supportive Care:** Continue supportive care measures that may include: Fluid Resuscitation: Ensure that adequate hydration has been maintained, especially if blood pressure was previously low. Monitoring: Close monitoring of vital signs, laboratory results (including lactate levels), and clinical status to detect any changes that may indicate a different diagnosis. Nutritional Support: Address nutrition as the patient recovers to support recovery.

4. **Consideration for Further Imaging or Testing:** If clinical suspicion remains high for a specific source of infection or other pathology, consider additional imaging studies or specific laboratory tests to rule out sources that may not have been initially apparent.

5. **Multidisciplinary Discussion:** Engage a multidisciplinary team, including infectious disease specialists, to discuss the case if the clinical picture remains complex or challenging to diagnose. Their insights can help refine the management approach based on current guidelines and practices.

6. **Follow-Up Plan:** Plan for follow-up assessments post-discharge to monitor the patient's recovery, ensuring appropriate outpatient care and addressing any persistent symptoms or complications.

Conclusion: In summary, the healthcare team should reassess the need for ongoing antibiotic treatment in the absence of a confirmed infectious source, continue supportive care, investigate alternative diagnoses, and involve specialists as needed to ensure comprehensive management of the patient's condition.

Score:

Comments:

Q16 (Complex/scenario-management) A 64-year-old woman is admitted to the medical ICU with possible community-acquired pneumonia. The patient is initiated on ceftriaxone and azithromycin. Chest radiography reveals a focal infiltrate. On admission, she is dyspneic with a respiratory rate of 33 breaths/minute. Her vital signs and laboratory values are as follows: blood pressure 90/50 mm Hg, heart rate 101 beats/minute, WBC 18 x 103 cells/mm3, and lactate 4.2 mmol/L. A PCT is obtained on admission. The results are available 12 hours after antibiotics are initiated. The PCT result is 0.1 mcg/L. What is your action regarding antibiotics?

Given the clinical scenario of the 64-year-old woman admitted with possible community-acquired pneumonia and initiated on ceftriaxone and azithromycin, the following considerations and recommended actions based on the Procalcitonin (PCT) result of 0.1 mcg/L (which indicates a low likelihood of a bacterial infection) are outlined:

Assessment of the Situation: Current Condition: The patient is presenting with signs of severe pneumonia and septic shock (elevated white blood cell count, hypotension, respiratory distress, and elevated lactate).

Antibiotics Initiated: Empiric antibiotic therapy (ceftriaxone and azithromycin) was commenced due to the suspicion of pneumonia.

PCT Result Interpretation: A low PCT level (0.1 mcg/L) typically suggests a low bacterial infection probability and can indicate that the infection may be viral or non-infectious in origin, especially in the context of pneumonia.

Recommended Action Regarding Antibiotics:

Continue Antibiotics for Now: Severe Illness & Initial Presentation: Despite the low PCT result, the patient presents with significant respiratory distress and indicators of severe sepsis, including hypotension and elevated lactate. It is crucial to continue antibiotic therapy for the time being due to the severity of her condition and the broader potential differential diagnoses related to pneumonia (which could include atypical or resistant pathogens).

Monitoring: Continuously monitor the patient's clinical status, vital signs, and laboratory markers closely to assess response to treatment and guide further management.

Pending Clinical Response: If the patient does not show improvement or there is deterioration observed over the next 24-48 hours, consider additional diagnostics to evaluate the cause of her symptoms.

This may include imaging studies, cultures, or possibly revisiting the treatment plan if no improvement is noted.

Re-evaluate in 24-48 Hours: Reassess the patient's clinical status and laboratory results within 24-48 hours, considering whether to de-escalate or switch antibiotics based on her clinical course, response to therapy, and further diagnostic results.

Engagement with Infectious Disease Specialist: Depending on the evolution of her clinical picture and response to treatment, consider involving an infectious disease specialist for further guidance, particularly if there is suspicion of non-bacterial causes or complications.

Conclusion: Given the patient's severe illness despite the low PCT result, it is prudent to continue the antibiotic therapy initially while closely monitoring her clinical condition and re-evaluating later based on her response to treatment and any new diagnostic findings. This approach balances diligence in treating a potentially life-threatening infection with the recognition of the low likelihood of bacterial involvement suggested by the PCT.

Score:

Comments:

Q17 78 years old male patient with medical history of diabetes mellitus, hypertension, previous abdominal surgery, and antibiotic exposure. Presented to the hospital with fever, chills, abdominal pain, tachycardia, hypotension, and confusion. Initially diagnosed with signs of septic shock, started fluid resuscitation and antimicrobials, including meropenem. What is your recommendation regarding this regimen?

Given the clinical scenario of the 78-year-old male patient presenting with signs consistent with septic shock and being treated with meropenem, the following recommendations should be considered regarding the antimicrobial regimen and overall management:

Recommendations:

Continue Empiric Antibiotic Therapy: Meropenem: This broad-spectrum carbapenem is an appropriate choice due to its efficacy against a wide range of gram-negative bacteria, including many resistant organisms, and it is particularly useful in patients with a history of previous antibiotic exposure and complicated medical history. Continue administering meropenem as part of the empiric therapy, especially in the setting of suspected intra-abdominal infection (e.g., peritonitis, abscess) related to his abdominal pain.

Additional Broad-Spectrum Coverage: Given the patient's history of previous antibiotic exposure, consider the potential for multidrug-resistant organisms. Depending on clinical suspicion for the source of infection (e.g., intra-abdominal vs. urinary tract), you may want to add coverage for MRSA (Methicillin-Resistant Staphylococcus aureus) if there is concern for skin or soft tissue infection, or if the patient is at

risk (e.g., previous infections, hospitalization). Vancomycin could be added in such cases. If there is a high suspicion of Pseudomonas or other resistant gram-negative bacteria, consider adding an aminoglycoside (e.g., gentamicin) or a different antibiotic that covers these pathogens, depending on rapid diagnostic results and local resistance patterns.

Reassessment after Cultures: Ensure that appropriate cultures (blood, urine, and any abdominal fluid if possible) are obtained before the initiation of antibiotics, as this aids in identifying specific pathogens. Adjust the antibiotic regimen based on culture results, sensitivity patterns, and clinical response once lab results return, potentially narrowing therapy to reduce antibiotic use while targeting the identified pathogens.

Monitor Patient Response: Closely monitor the patient's clinical status (vital signs, consciousness, laboratory values like lactate levels), response to fluid resuscitation, and effectiveness of empirical antibiotics. If there is no improvement in clinical status within 48 hours, consider reassessing the source of infection for possible surgical or interventional management.

Infection Source Control: Consult surgical or interventional teams if there are any indications of an intra-abdominal source (e.g., abscess, perforation) that may require drainage or surgical intervention.

Consideration of Duration of Therapy: Generally, for sepsis, a duration of 7 to 10 days is recommended, depending on the clinical scenario and source of infection. Reassess regularly in the context of improving patient condition, laboratory markers, and whether the infection source has been effectively managed.

Conclusion: In this critical scenario, the antimicrobial regimen of meropenem appears appropriate as part of the broad-spectrum empirical therapy, potentially supplemented with additional agents based on specific clinical concerns or local resistance patterns. Regular monitoring and reassessment remain key components in effectively managing this patient with septic shock while minimizing potential antibiotic resistance and adverse effects.

Score:

Comments:

Q18 67-year-old male, known case of Hypertension, type 2 Diabetes, Chronic Obstructive Pulmonary Disease (COPD). Admitted to the ICU 48 hours ago following a severe case of community-acquired pneumonia resulting in respiratory failure. Currently intubated and mechanically ventilated, sedated, and on vasopressors to maintain blood pressure. Current Medications: Vasopressors (Norepinephrine), Sedation (Propofol), Antibiotics (Ceftriaxone and Azithromycin), Insulin sliding scale for glucose management, Vital Signs: Blood Pressure 100/60 mmHg on vasopressors, Heart Rate 85 bpm, Respiratory Rate 18 bpm (via ventilator). Lab Results: Elevated inflammatory markers, creatinine is

stable at 1.1 mg/dL. What are the possible approaches regarding VTE (Venous Thromboembolism) prophylaxis?

For a 67-year-old male patient in the ICU with multiple risk factors, including hypertension, type 2 diabetes, chronic obstructive pulmonary disease (COPD), respiratory failure, and prolonged immobility due to intubation, it is crucial to establish appropriate strategies for VTE (Venous Thromboembolism) prophylaxis. Here's an overview of possible approaches:

Approaches for VTE Prophylaxis:

Pharmacologic Prophylaxis:

- **Low-Molecular-Weight Heparin (LMWH):** Consider administering enoxaparin (Lovenox) as a standard pharmacologic prophylactic option unless contraindicated (e.g., active bleeding or high risk of bleeding). The typical dosing is usually 40 mg subcutaneously once daily or 30 mg every 12 hours for patients with impaired renal function (creatinine clearance < 30 mL/min).
- **Unfractionated Heparin (UFH):** In cases where there are concerns of renal impairment, bleeding risks, or if the patient is on multiple medications that could interact with LMWH, consider using unfractionated heparin instead. Dosing for prophylaxis would typically be 5,000 units subcutaneously every 8-12 hours.

Monitoring: Regularly monitor the patient's platelet levels and signs of bleeding if on anticoagulation therapy.

Mechanical Prophylaxis:

If there are contraindications to pharmacologic prophylaxis (such as active bleeding, recent major surgery, or thrombocytopenia), use mechanical prophylactic measures:

Sequential Compression Devices (SCDs): Apply SCDs on both legs to promote venous return and reduce stasis. These devices should be used continuously while the patient is in bed.

Intermittent Pneumatic Compression: This method can also be considered to facilitate venous flow, particularly for patients at high risk of VTE.

Combined Approaches: If the patient can safely receive pharmacologic prophylaxis, a combination of both pharmacologic and mechanical methods can be employed, especially in higher-risk patients. For example, administer LMWH or UFH while using SCDs for additional protection.

Patient Mobility: Involve physical therapy or nursing to encourage early mobilization as tolerated. Initiating passive or active range-of-motion exercises can help mitigate the risk of VTE, although this may be limited due to the patient being intubated and sedated.

Re-evaluate Risk Factors: Continuously assess the patient's risk factors for VTE over time as clinical conditions change. Consider re-evaluating the need for continued anticoagulation if there are changes in the patient's status (e.g., improvement in respiratory function allowing for extubation or increased mobility).

Consider Duration of Prophylaxis: Initiate VTE prophylaxis as soon as feasible after admission to the ICU and continue for the duration of hospitalization, especially throughout the period of immobility. Reassess the need for extended prophylaxis after discharge, particularly in light of ongoing risk factors.

Conclusion:

Implementing an effective VTE prophylaxis strategy in this patient should primarily involve pharmacologic prophylaxis using LMWH or UFH as appropriate, along with mechanical measures like SCDs when feasible, while also monitoring the patient's evolving clinical status and risk factors. It is vital to strike a balance between providing optimal prophylaxis and minimizing the risk of complications from anticoagulation therapy.

Score:

Comments:

End of Questions-AI(1)-Platform.
