# Supplementary material for: Evaluating the Accuracy of Medical Information Generated by ChatGPT and Gemini and Its Alignment With International Clinical Guidelines From the Surviving Sepsis Campaign: Comparative Study
Source: JMIR Form Res. 2025 Dec 17;9:e84251. doi: 10.2196/84251 (PMC12756662; doi:10.2196/84251)
Supplement: Multimedia Appendix 3 [file formative_v9i1e84251_app3.pdf]

Q1 What are effective strategies for hospitals to enhance the early detection and treatment of sepsis and septic shock in a timely manner?

Hospitals can enhance the early detection and treatment of sepsis and septic shock by implementing the following strategies:

Early Recognition and Rapid Response: Implement sepsis screening tools to identify patients at risk. Establish clear protocols for early recognition and rapid response to suspected sepsis. Train healthcare providers on sepsis recognition and management. Use electronic health records to track vital signs and laboratory results to identify early warning signs.

Rapid Diagnostic Testing: Prioritize rapid diagnostic tests for sepsis, such as blood cultures and lactate levels. Consider using point-of-care tests for rapid assessment of inflammatory markers.

Early and Aggressive Treatment: Administer broad-spectrum antibiotics within one hour of sepsis recognition. Initiate rapid fluid resuscitation to restore blood pressure and organ perfusion. Consider using vasopressors to maintain blood pressure if fluids alone are insufficient. Monitor blood glucose levels and administer insulin if necessary to maintain glucose control. Implement early source control measures, such as drainage of infected fluids or surgical removal of infected tissue.

Continuous Monitoring and Evaluation: Continuously monitor vital signs, laboratory results, and clinical status of patients with sepsis. Adjust treatment as needed based on patient response and evolving clinical picture. Implement regular quality improvement initiatives to track sepsis outcomes and identify areas for improvement.

Multidisciplinary Collaboration: Foster collaboration between physicians, nurses, pharmacists, and other healthcare professionals to ensure timely and coordinated care. Establish a dedicated sepsis team to oversee patient care and quality improvement efforts.

By implementing these strategies, hospitals can significantly improve the early detection and treatment of sepsis and septic shock, leading to better patient outcomes and reduced mortality rates.

Score:

Comments:

Q2 What is the initial recommended crystalloid fluid of choice for sepsis resuscitation? Mention the full strategy?

The initial crystalloid fluid of choice for sepsis resuscitation is balanced crystalloid solution, such as lactated Ringer's solution. This is preferred over normal saline due to its more balanced electrolyte profile, which helps to minimize electrolyte disturbances and potential harm to the kidneys.

The full strategy for sepsis resuscitation includes the following:

Early Recognition and Rapid Response:

- Implement sepsis screening tools to identify patients at risk.
- Establish clear protocols for early recognition and rapid response to suspected sepsis.
- Train healthcare providers on sepsis recognition and management.
- Use electronic health records to track vital signs and laboratory results to identify early warning signs.

Rapid Diagnostic Testing:

- Prioritize rapid diagnostic tests for sepsis, such as blood cultures and lactate levels.
- Consider using point-of-care tests for rapid assessment of inflammatory markers.

Early and Aggressive Treatment:

- Administer broad-spectrum antibiotics within one hour of sepsis recognition.
- Initiate rapid fluid resuscitation with balanced crystalloid solutions to restore blood pressure and organ perfusion.
- Consider using vasopressors to maintain blood pressure if fluids alone are insufficient.
- Monitor blood glucose levels and administer insulin if necessary to maintain glucose control.
- Implement early source control measures, such as drainage of infected fluids or surgical removal of infected tissue.

Continuous Monitoring and Evaluation:

- Continuously monitor vital signs, laboratory results, and clinical status of patients with sepsis.
- Adjust treatment as needed based on patient response and evolving clinical picture.
- Implement regular quality improvement initiatives to track sepsis outcomes and identify areas for improvement.

Multidisciplinary Collaboration: Foster collaboration between physicians, nurses, pharmacists, and other healthcare professionals to ensure timely and coordinated care. Establish a dedicated sepsis team to oversee patient care and quality improvement efforts.

Score: **Comments:**

Q3 What is the approach to start antimicrobial therapy when sepsis is recognized?

This is for informational purposes only. For medical advice or diagnosis, consult a professional.

When sepsis is recognized, the approach to start antimicrobial therapy is as follows:

- **Obtain Cultures:** Before administering antibiotics, obtain blood cultures and any other relevant cultures (e.g., urine, sputum, wound) to identify the specific pathogen causing the infection.
- **Empiric Therapy:** While waiting for culture results, initiate empiric broad-spectrum antibiotic therapy to cover a wide range of potential pathogens. The choice of antibiotics will depend on the suspected source of infection, the patient's risk factors, and local resistance patterns.
- **De-escalation:** Once culture results are available, the antibiotic regimen can be de-escalated to a narrower-spectrum antibiotic that is specifically targeted against the identified pathogen.
- **Duration of Therapy:** The duration of antibiotic therapy will depend on the severity of the infection, the specific pathogen involved, and the patient's clinical response.

It is important to note that the timing of antibiotic administration is critical in the management of sepsis. Early administration of appropriate antibiotics within the first hour of sepsis recognition is associated with improved patient outcomes.

Score:

Comments:

Q4 What is the approach to address source control in sepsis and septic shock?

This is for informational purposes only. For medical advice or diagnosis, consult a professional.

Source control is a crucial aspect of sepsis and septic shock management. It involves identifying and addressing the underlying source of infection to reduce the bacterial load and promote healing. Here's a general approach to source control:

Identification of the Source:

Thorough Clinical Evaluation: A comprehensive physical examination is essential to identify potential sources of infection, such as:

- Intra-abdominal: Abscesses, peritonitis, diverticulitis, pancreatitis
- Pulmonary: Pneumonia, lung abscess
- Urinary Tract: Urinary tract infection, pyelonephritis
- Skin and Soft Tissue: Cellulitis, necrotizing fasciitis, wound infections
- Vascular: Endocarditis, septic thrombophlebitis

- Central Line-Associated: Catheter-related bloodstream infection

Diagnostic Tests: Imaging studies (X-rays, CT scans, ultrasound) and laboratory tests (blood cultures, urine cultures) may be necessary to confirm the source of infection.

Source Control Strategies:

- Surgical Intervention: Surgical intervention is often required to drain abscesses, remove infected foreign bodies, or debride necrotic tissue. This may involve procedures such as:
- Percutaneous drainage: Using a needle or catheter to drain abscesses under imaging guidance.
- Open surgical drainage: Involving incision and drainage of abscesses or infected tissues.
- Debridement: Removal of necrotic or infected tissue.
- Source removal: Removal of infected devices, such as catheters or prosthetic materials.
- Non-Surgical Interventions: In some cases, non-surgical interventions may be sufficient, such as:

Antibiotic therapy: To target the specific pathogen causing the infection.

Catheter removal: Removing unnecessary catheters to reduce the risk of infection.

Wound care: Proper cleaning and dressing of wounds to promote healing.

Timing of Source Control:

Early Source Control: Early identification and treatment of the source of infection are crucial for improving patient outcomes.

Balancing Risks and Benefits: In some cases, delaying source control may be necessary to stabilize the patient's condition before undergoing surgery.

Multidisciplinary Approach:

Collaboration: A multidisciplinary team, including infectious disease specialists, surgeons, intensivists, and other healthcare professionals, should work together to optimize source control strategies.

Remember, the specific approach to source control will vary depending on the individual patient's condition and the identified source of infection. Timely and effective source control is essential for improving patient outcomes in sepsis and septic shock.

Score:

Comments:

Q5 What are the recommended strategies for managing vasoactive drugs (epinephrine, norepinephrine, vasopressin, dopamine, dobutamine, terlipressin and levosimendan) in septic shock?

This is for informational purposes only. For medical advice or diagnosis, consult a professional.

Managing vasoactive drugs in septic shock requires a careful and individualized approach. Here are some general strategies: **Early Goal-Directed Therapy (EGDT):** This approach involves early and aggressive fluid resuscitation, followed by the use of vasopressors to maintain mean arterial pressure (MAP) at 65-70 mmHg.

Choice of Vasopressor:

- Norepinephrine: This is the first-line vasopressor for most patients with septic shock due to its potent alpha-adrenergic vasoconstrictor effects and minimal beta-adrenergic effects.
- Epinephrine: This can be considered as a second-line agent, especially in patients with refractory hypotension or myocardial dysfunction.
- Vasopressin: This can be added as a second-line agent in patients with persistent hypotension despite adequate fluid resuscitation and norepinephrine therapy.
- Dopamine: This has a complex action and is not recommended as a first-line agent.
- Dobutamine: This is an inotropic agent that can be used to improve cardiac output in patients with myocardial dysfunction.
- Terlipressin and Levosimendan: These agents have limited data in septic shock and are not routinely recommended.

Titration: Vasoactive drugs should be titrated to achieve the desired hemodynamic goals, with frequent monitoring of blood pressure, heart rate, and urine output.

Continuous Monitoring: Close monitoring of hemodynamic parameters, electrolyte balance, and organ function is essential.

Combination Therapy: In some cases, a combination of vasopressors and inotropes may be necessary to achieve optimal hemodynamic support.

Weaning: Once hemodynamic stability is achieved, the dose of vasoactive drugs should be gradually reduced and eventually discontinued.

It's important to note that the management of vasoactive drugs in septic shock is complex and should be guided by experienced clinicians. Individual patient factors, such as underlying comorbidities and hemodynamic status, should be considered when making treatment decisions.

Score:

Comments:

Q6 Under which circumstances should corticosteroids be used in septic shock?

This is for informational purposes only. For medical advice or diagnosis, consult a professional.

Corticosteroids are recommended for use in septic shock under the following circumstances:

Refractory Hypotension: Patients who remain hypotensive despite adequate fluid resuscitation and vasopressor therapy may benefit from corticosteroids. This is because corticosteroids can help to improve vascular tone and reduce systemic inflammation.

Adrenal Insufficiency: Patients with suspected or confirmed adrenal insufficiency should receive corticosteroids to supplement their deficient cortisol production. Adrenal insufficiency can worsen hemodynamic instability in septic shock.

Severe Sepsis with Organ Dysfunction: Some studies suggest that corticosteroids may improve organ function and reduce mortality in patients with severe sepsis and multiple organ dysfunction syndrome (MODS).

It is important to note that the use of corticosteroids in septic shock is controversial, and their benefits must be weighed against potential risks, such as hyperglycemia, immunosuppression, and increased risk of infection. Corticosteroids should only be used under the supervision of experienced clinicians, and their use should be carefully monitored.

Score:

Comments:

Q7 What is the approach of using neuromuscular blocking agents in adults' sepsis-induced ARDS?

This is for informational purposes only. For medical advice or diagnosis, consult a professional.

Neuromuscular blocking agents (NMBAs) can be a valuable tool in the management of sepsis-induced acute respiratory distress syndrome (ARDS). By paralyzing the patient, NMBAs can help to improve ventilator synchrony, reduce ventilator-induced lung injury (VILI), and improve oxygenation.

However, the use of NMBAs in ARDS is controversial, and their use should be carefully considered on a case-by-case basis. Here's the approach to using NMBAs in sepsis-induced ARDS:

Indications: Severe ARDS: NMBAs are primarily indicated for patients with severe ARDS who are difficult to ventilate despite optimal ventilator settings and sedation. **Refractory Hypoxemia:** Patients who remain hypoxemic despite aggressive ventilation strategies may benefit from NMBAs.

Contraindications:

- Muscle Weakness: Patients with preexisting muscle weakness or neuromuscular disorders should avoid NMBAs.
- Electrolyte Imbalances: Severe electrolyte imbalances, such as hypokalemia or hypocalcemia, can increase the risk of NMBA-related complications.

Monitoring:

- Continuous Monitoring: Close monitoring of hemodynamic parameters, oxygenation, ventilation, and neuromuscular function is essential during NMBA therapy.
- Sedation: Adequate sedation should be provided to minimize patient discomfort and anxiety.
- Electrolyte Balance: Electrolyte levels should be closely monitored and corrected as needed.
- Muscle Strength: Regular assessment of muscle strength is important to identify potential muscle weakness and guide weaning from NMBAs.

Weaning:

Gradual Weaning: Once the patient is hemodynamically stable and oxygenation is adequate, NMBAs can be gradually weaned. Close Monitoring: Close monitoring is essential during the weaning process to identify any signs of respiratory distress or muscle weakness.

It is important to note that the use of NMBAs in ARDS is a complex issue, and the decision to use NMBAs should be made on an individual basis by experienced clinicians. While NMBAs can be a valuable tool in the management of severe ARDS, they should be used with caution and with appropriate monitoring.

Score:

Comments:

Q8 What are the recommendations regarding the routine use Bicarbonate therapy and IV vitamin C in sepsis and septic shock?

This is for informational purposes only. For medical advice or diagnosis, consult a professional.

Bicarbonate Therapy:

- **Limited Evidence:** While bicarbonate therapy was once a common practice in sepsis and septic shock, current evidence does not support its routine use.
- **Potential Risks:** Bicarbonate therapy can lead to alkalosis, which may worsen tissue perfusion and myocardial function.
- **Indications:** Bicarbonate therapy may be considered in specific circumstances, such as severe metabolic acidosis with a pH below 7.2 and evidence of impaired tissue perfusion. However, this decision should be made on a case-by-case basis and in consultation with a specialist.

IV Vitamin C:

- **Emerging Evidence:** Recent studies have shown promising results with the use of intravenous vitamin C in sepsis and septic shock. Vitamin C may help to reduce oxidative stress, improve immune function, and enhance vascular stability.
- **Current Recommendations:** While the evidence is still evolving, some guidelines suggest that IV vitamin C may be considered as an adjunct therapy in patients with severe sepsis and septic shock, especially those with persistent hypotension.
- **Further Research:** More large-scale, randomized controlled trials are needed to definitively establish the role of IV vitamin C in sepsis management.

It's important to note that the use of both bicarbonate therapy and IV vitamin C should be guided by experienced clinicians and based on individual patient factors. These therapies should not be used as a substitute for standard sepsis management practices, such as early recognition, rapid antibiotic administration, and aggressive fluid resuscitation.

Score:

Comments:

Q9 What are the approaches for nutrition and glycemic control in patient with sepsis and septic shock?

Nutrition in Sepsis and Septic Shock:

Early nutritional support is crucial for patients with sepsis and septic shock. It helps to maintain protein synthesis, immune function, and organ function. Here's a general approach:

- **Early Initiation:** Nutritional support should be initiated within 24-48 hours of illness onset, even if the patient is initially unable to tolerate oral intake.
- **Enteral Nutrition:** Enteral nutrition is preferred over parenteral nutrition whenever possible, as it helps to maintain gut integrity and reduce the risk of infection.
- **Parenteral Nutrition:** If enteral nutrition is not feasible, parenteral nutrition can be provided to meet the patient's nutritional needs.
- **Caloric and Protein Requirements:** The caloric and protein requirements of patients with sepsis and septic shock are often increased. A registered dietitian can help to calculate these needs and develop an appropriate nutrition plan.

Monitoring: Close monitoring of nutritional intake, weight, and laboratory parameters is essential to ensure adequate nutritional support.

Glycemic Control in Sepsis and Septic Shock

Tight glycemic control is important in sepsis and septic shock to reduce the risk of complications such as infection and organ dysfunction. Here's a general approach:

- **Target Blood Glucose:** The target blood glucose range is typically 140-180 mg/dL.
- **Insulin Therapy:** Insulin therapy may be required to achieve tight glycemic control.
- **Continuous Glucose Monitoring:** Continuous glucose monitoring can help to optimize insulin therapy and minimize the risk of hypoglycemia.

Monitoring: Close monitoring of blood glucose levels is essential to avoid hypoglycemia and hyperglycemia.

By implementing these strategies, healthcare providers can improve the nutritional status and glycemic control of patients with sepsis and septic shock, leading to better outcomes.

Score:

Comments:

Q10 65-year-old man who presented with symptoms of sepsis. He was admitted with high fever, tachycardia, low blood pressure, and confusion. Blood cultures are pending, but his clinical presentation strongly suggests septic shock, requiring immediate resuscitation. The attending physician is briefing the team on the optimal management strategy. The discussion focuses on the choice of intravenous fluid for resuscitation to restore his hemodynamic status. As the team explores different fluid options including crystalloids, albumin, starches, and synthetic colloids, such as gelatin. What will be the best choice for him?

In this scenario, the best initial fluid choice for resuscitation would be a balanced crystalloid solution, such as Lactated Ringer's solution.

Here's why:

- Rapid Volume Expansion: Crystalloids are readily available, easily administered, and rapidly expand the intravascular volume.
- Electrolyte Balance: Balanced crystalloids, like Lactated Ringer's, closely mimic the body's electrolyte composition, minimizing electrolyte disturbances.
- Cost-Effective: Crystalloids are generally more cost-effective than colloid solutions.

While colloid solutions (like albumin or synthetic colloids) can offer advantages in certain situations, they are not typically the first-line choice for initial resuscitation in septic shock.

Key Considerations:

- **Continuous Monitoring:** Close monitoring of hemodynamic parameters (blood pressure, heart rate, urine output) is essential to guide fluid therapy.
- **Avoid Fluid Overload:** Excessive fluid administration can lead to pulmonary edema and other complications.
- **Consider Vasopressors:** If fluid resuscitation alone is insufficient to maintain adequate blood pressure, vasopressors may be required.

It's important to note that the specific fluid therapy strategy should be tailored to the individual patient's clinical condition and response to treatment.

Score:

Comments:

Q11 A 58-year-old woman was admitted to the ICU with severe sepsis due to a urinary tract infection. She required mechanical ventilation for five days and spent ten days in the ICU. After a successful treatment, she is now stable and ready for discharge from the hospital. What is your plan for follow-up?

Follow-up Plan for a Patient Recovering from Severe Sepsis, A comprehensive follow-up plan is essential for patients recovering from severe sepsis to monitor their recovery, address potential complications, and prevent future infections. Here's a suggested approach:

Immediate Post-Discharge Follow-Up:

- Within 1-2 Weeks: Schedule a follow-up appointment with the primary care physician or infectious disease specialist.
- Assess physical and cognitive function
- Evaluate wound healing and any surgical site infections.
- Review medication adherence and side effects.
- Provide education on infection prevention strategies, including hand hygiene and recognizing signs of infection.

Long-Term Follow-Up:

- Regular Check-ups: Schedule regular check-ups with the primary care physician, typically every 3-6 months for the first year. Frequency of visits can be adjusted based on the patient's specific needs and risk factors.

Monitoring for Complications: Screen for potential long-term complications of sepsis, such as:

- Chronic fatigue
- Post-intensive care syndrome (PICS)
- Organ dysfunction
- Mental health issues (anxiety, depression)
- Monitor for recurrent infections, especially urinary tract infections.

Lifestyle Modifications:

- Encourage a healthy lifestyle, including a balanced diet, regular exercise, and adequate sleep.
- Advise on smoking cessation and alcohol moderation.

Vaccinations: Ensure up-to-date vaccinations, including influenza and pneumococcal vaccines.

Additional Considerations:

- **Referrals:** Refer the patient to appropriate specialists, such as a pulmonologist, cardiologist, or nephrologist, if necessary.
- **Social Support:** Assess the patient's social support system and connect them with appropriate resources, such as home health services or social workers.
- **Patient Education:** Provide clear and concise information about the patient's condition, treatment plan, and follow-up care.

By implementing a comprehensive follow-up plan, healthcare providers can help patients recover from severe sepsis and minimize the risk of long-term complications.

Score:

Comments:

Q12 72-year-old male diagnosed with septic shock, suspected to be due to a gram-negative infection with risk factors of Recent hospitalization, prior antibiotic exposure, and a past history of urinary tract infections resistant to first-line antibiotics. ICU doctors started him on meropenem and ciprofloxacin plus vancomycin. What are your recommendations regarding antibiotic choices?

This is for informational purposes only. For medical advice or diagnosis, consult a professional.

Assessing the Antibiotic Regimen: The initial antibiotic regimen chosen for this patient, including meropenem, ciprofloxacin, and vancomycin, is a reasonable approach given the patient's clinical presentation and risk factors. However, a few considerations should be made:

1. Gram-Negative Coverage:

- Meropenem: This broad-spectrum carbapenem is an excellent choice for suspected gram-negative infections, including those resistant to first-line antibiotics. It covers a wide range of gram-negative organisms, including Pseudomonas aeruginosa and Acinetobacter baumannii.

- Ciprofloxacin: While ciprofloxacin can be effective against certain gram-negative organisms, its coverage is more limited compared to meropenem. It may be considered as adjunctive therapy, especially if there is a concern for specific resistant organisms.

2. Gram-Positive Coverage:

- Vancomycin: This is a suitable choice for empiric coverage against methicillin-resistant Staphylococcus aureus (MRSA). However, given the patient's clinical presentation and risk factors, the likelihood of MRSA infection is relatively low.

Recommendations:

Re-evaluate Antibiotic Coverage: Once culture results are available, the antibiotic regimen should be de-escalated to the narrowest spectrum of antibiotics that can effectively treat the identified pathogen. If the culture results reveal a gram-negative organism susceptible to meropenem, the ciprofloxacin can be discontinued. If MRSA is not identified, vancomycin can be discontinued.

Consider Antibiotic Susceptibility Testing:

Perform susceptibility testing on the isolated pathogen to guide optimal antibiotic therapy. If the organism is resistant to meropenem, alternative antibiotics, such as piperacillin-tazobactam or ceftazidime-avibactam, may be considered.

Infection Control Measures:

Implement strict infection control measures to prevent the spread of resistant organisms. Adhere to hand hygiene guidelines and proper isolation procedures.

Close Monitoring:

Monitor the patient's clinical response to therapy, including vital signs, laboratory parameters, and organ function. Adjust the antibiotic regimen as needed based on the patient's response and culture results.

By following these recommendations, healthcare providers can optimize antibiotic therapy for this patient, minimize the risk of adverse effects, and improve clinical outcomes.

Score:

Comments:

Q13 58-year-old female patient in the ICU. She was recently diagnosed with severe acute respiratory distress syndrome (ARDS) as a complication of sepsis secondary to community-acquired pneumonia. She is on mechanical ventilation, and you are part of the multidisciplinary team responsible for managing her ventilatory settings to optimize her respiratory function while minimizing further lung injury. What are the key elements of ventilatory management that can prevent ventilator-induced lung injury? Mention the full strategy

This is for informational purposes only. For medical advice or diagnosis, consult a professional. Key Elements of Ventilatory Management to Prevent Ventilator-Induced Lung Injury (VILI) in ARDS:

- **Low Tidal Volume Ventilation (LTVV):** Rationale: LTVV aims to minimize alveolar overdistension and reduce the risk of ventilator-induced lung injury.
- **Target Tidal Volume:** Aim for a tidal volume of 6 ml/kg predicted body weight.

- **Positive End-Expiratory Pressure (PEEP):** Rationale: PEEP helps to maintain alveolar distension and improve oxygenation.
- **PEEP Setting:** The optimal level of PEEP varies depending on the individual patient. Generally, a PEEP level of 5-15 cm H2O is recommended.
- **PEEP Titration:** PEEP can be titrated based on the patient's oxygenation status, plateau pressure, and hemodynamic stability.
- **Limiting Plateau Pressure:** Rationale: High plateau pressures can lead to alveolar overdistension and barotrauma.
- **Target Plateau Pressure:** The target plateau pressure is typically <30 cm H2O.
- **Permissive Hypercapnia:** Rationale: Allowing a modest increase in PaCO2 can help to reduce tidal volume and minimize lung injury.
- **Target PaCO2:** A PaCO2 of 45-55 mmHg is generally acceptable in patients with ARDS.

Lung-Protective Ventilation Strategies:

- **Recruitment Maneuvers:** Occasional application of high PEEP (30-40 cm H2O) for a short period can help to recruit collapsed lung units.
- **Prone Positioning:** Prone positioning can improve oxygenation and reduce the risk of VILI.
- **Sedation and Analgesia:** Rationale: Adequate sedation and analgesia can help to minimize patient-ventilator desynchrony and reduce the work of breathing.
- **Sedation Strategy:** Use light sedation to allow for frequent neurologic assessments and early weaning from the ventilator.

Monitoring and Adjustments: Continuous Monitoring: Closely monitor the patient's respiratory parameters, including respiratory rate, tidal volume, PEEP, FiO2, PaO2, PaCO2, and hemodynamic parameters.

Frequent Adjustments: Adjust ventilator settings as needed to optimize oxygenation and minimize lung injury.

Additional Considerations:

- Early Liberation from Mechanical Ventilation: Aim to wean the patient from mechanical ventilation as soon as possible to reduce the risk of ventilator-associated complications.
- Non-Invasive Ventilation: Consider non-invasive ventilation (NIV) as an alternative to invasive mechanical ventilation in appropriate patients.
- Nutrition: Adequate nutritional support is essential for lung repair and overall recovery.
- Infection Prevention: Implement strict infection control measures to prevent ventilator-associated pneumonia.

By implementing these strategies, healthcare providers can optimize ventilator management and reduce the risk of VILI in patients with ARDS.

Score:

Comments:

Q14 60-year-old female, type 2 diabetes, arrives at the hospital with complaints of severe fatigue, shortness of breath, and a productive cough. In the emergency department, her vital signs are as follows: temperature 38.3°C (100.9°F), heart rate 105 beats per minute, respiratory rate 24 breaths per minute, and blood pressure 118/70 mmHg. Her oxygen saturation is 92% on room air. The medical team considers assessing her for possible sepsis. The quick Sequential Organ Failure Assessment (qSOFA) score, based on altered mentation, low blood pressure, and increased respiratory rate, is considered as part of the initial screening process. What might be the limitations of relying solely on the qSOFA score for identifying sepsis? Give a rational compared to other assessment tools

This is for informational purposes only. For medical advice or diagnosis, consult a professional.

Limitations of relying solely on the qSOFA score for identifying sepsis:

Low sensitivity: qSOFA has a low sensitivity, meaning it may miss a significant number of patients with sepsis (false negatives). This is particularly concerning in early sepsis, when prompt treatment is crucial.

Limited to a few organ systems: qSOFA only assesses three organ systems: respiratory, neurological, and cardiovascular. Sepsis can affect multiple organ systems, and relying solely on qSOFA may miss other signs of organ dysfunction.

Does not assess underlying infection: qSOFA does not assess the presence of an underlying infection, which is a key component of sepsis. This can lead to missed diagnoses, especially in patients with atypical presentations.

May not be applicable in certain populations: qSOFA was developed and validated in critically ill patients. Its accuracy in other patient populations, such as older adults or those with chronic comorbidities, may be limited.

Other assessment tools for sepsis:

Sequential Organ Failure Assessment (SOFA) score: This is a more comprehensive score that assesses six organ systems: respiratory, cardiovascular, neurological, hepatic, renal, and coagulation. It is more sensitive than qSOFA but requires more resources and time to calculate.

Systemic Inflammatory Response Syndrome (SIRS) criteria: This assesses four physiological parameters: temperature, heart rate, respiratory rate, and white blood cell count. It is easy to calculate but has low specificity, meaning it may identify many patients without sepsis (false positives).

National Early Warning Score (NEWS): This is a tool used in the UK to assess the overall risk of deterioration in hospitalized patients. It includes multiple physiological parameters and can be used to identify patients at risk of sepsis.

Clinical judgment: The most important tool for identifying sepsis is clinical judgment. Experienced clinicians can assess a patient's overall clinical picture, including their history, physical exam, and vital signs, to identify signs of sepsis and other organ dysfunction.

In conclusion, qSOFA is a useful screening tool for identifying patients at risk of sepsis, but it should not be used in isolation. Other assessment tools, such as SOFA, SIRS, NEWS, and clinical judgment, should be used in conjunction with qSOFA to improve the accuracy of sepsis diagnosis.

Score:

Comments:

Q15 52-year-old gentleman with a history of hypertension presents to the emergency department with high fever, chills, and confusion. On physical examination, he appears acutely ill with a temperature of 38.9°C (102°F), a heart rate of 112 beats per minute, and a blood pressure of 86/58 mmHg. Initial blood tests reveal an elevated white blood cell count and elevated lactate levels, suggesting possible sepsis. However, blood and urine cultures are negative after 48 hours, and no clear source of infection is identified. The medical team suspects sepsis or septic shock and initiates empiric broad-spectrum antibiotics. Over the next 24 hours, his condition stabilizes, but no evidence of bacterial infection is found from subsequent tests and imaging. Considering the recommendation on empiric antimicrobials, what should the healthcare team consider doing next in managing his treatment?

Considering the negative culture results and the stabilization of the patient's condition, the healthcare team should consider the following steps:

De-escalation of Antibiotics: Since no definitive source of infection has been identified, and the patient's condition is improving, the empiric antibiotic therapy should be de-escalated or discontinued. This decision should be made in consultation with an infectious disease specialist.

Investigate Other Potential Causes of Sepsis-Like Illness:

- **Viral Infections:** Consider testing for viral infections, such as influenza, respiratory syncytial virus (RSV), or COVID-19.
- **Fungal Infections:** In immunocompromised patients or those with prolonged hospital stays, fungal infections (e.g., Candida, Aspergillus) should be considered.
- **Non-Infectious Causes:** Other potential causes of sepsis-like illness include inflammatory bowel disease, pancreatitis, or autoimmune disorders.
- **Monitor for Complications:** Continue to monitor the patient's vital signs, organ function, and mental status. Be vigilant for potential complications of sepsis, such as acute kidney injury, acute respiratory distress syndrome (ARDS), or disseminated intravascular coagulation (DIC).
- **Supportive Care:** Continue to provide supportive care, including fluid resuscitation, vasopressor support (if needed), and pain management. Consider early mobilization and nutritional support to promote recovery.

Important Considerations:

- Antibiotic Stewardship: It is crucial to use antibiotics judiciously to prevent the emergence of antibiotic-resistant bacteria.
- Individualized Approach: The management of sepsis should be tailored to the individual patient's clinical presentation, risk factors, and response to therapy.

- Multidisciplinary Approach: A multidisciplinary team, including infectious disease specialists, critical care physicians, and other healthcare professionals, should collaborate to optimize patient care.

By following these recommendations, the healthcare team can effectively manage this patient's condition, minimize the risk of complications, and improve patient outcomes.

Score:

Comments:

**Q16 A 64-year-old woman is admitted to the medical ICU with possible community-acquired pneumonia. The patient is initiated on ceftriaxone and azithromycin. Chest radiography reveals a focal infiltrate. On admission, she is dyspneic with a respiratory rate of 33 breaths/minute. Her vital signs and laboratory values are as follows: blood pressure 90/50 mm Hg, heart rate 101 beats/minute, WBC 18 x 103 cells/mm3, and lactate 4.2 mmol/L. A PCT is obtained on admission. The results are available 12 hours after antibiotics are initiated. The PCT result is 0.1 mcg/L. What is your action regarding
antibiotics?**

Assessing the PCT Result and Adjusting Antibiotic Therapy

- **Understanding PCT:** Procalcitonin (PCT) is a biomarker that can help differentiate between bacterial and viral infections. A low PCT level, as in this case (0.1 mcg/L), typically indicates a low probability of a severe bacterial infection.
- **Decision-Making:** Given the low PCT level and the patient's clinical improvement (assuming she is improving), the following steps can be considered:

De-escalation of Antibiotics:

- **Rationale:** The low PCT level suggests that the initial broad-spectrum antibiotic therapy may be excessive.
- **Action:** Consider de-escalating the antibiotic regimen to a narrower-spectrum agent, such as oral antibiotics, or discontinuing antibiotics altogether.

Close Monitoring: Continue to monitor the patient's clinical status, vital signs, and laboratory parameters. Watch for any signs of worsening infection or development of new complications.

Repeat PCT:

- If the patient's clinical condition deteriorates or there is uncertainty about the adequacy of antibiotic therapy, consider repeating the PCT measurement.
- A rising PCT level may indicate persistent or worsening infection.

Additional Considerations:

- **Patient-Specific Factors:** The decision to de-escalate or discontinue antibiotics should be individualized, considering the patient's overall clinical status, risk factors, and comorbidities.
- **Local Antibiotic Resistance Patterns:** The local epidemiology of antibiotic resistance should be considered when making decisions about antibiotic therapy.

- **Consultation with Infectious Disease Specialist:** Consulting with an infectious disease specialist can provide expert guidance on antibiotic stewardship and infection control.

By following these guidelines and closely monitoring the patient's clinical course, healthcare providers can optimize antibiotic therapy, minimize the risk of adverse effects, and promote patient recovery.

Score:

Comments:

Q17 78-year-old male patient with medical history of diabetes mellitus, hypertension, previous abdominal surgery, and antibiotic exposure. Presented to the hospital with fever, chills, abdominal pain, tachycardia, hypotension, and confusion. Initially diagnosed with signs of septic shock, started fluid resuscitation and antimicrobials, including meropenem. What is your recommendation regarding this regimen?

The initial decision to start meropenem for a patient with suspected sepsis is reasonable, especially considering the patient's risk factors for multidrug-resistant organisms (MDROs). However, a few factors should be considered:

1. Broad-Spectrum Coverage: Meropenem is a broad-spectrum antibiotic that covers a wide range of Gram-negative bacteria, including many MDR organisms. However, it's essential to consider the potential for antibiotic-associated adverse effects, including Clostridium difficile infection.

2. Source Control: Identifying and addressing the underlying source of infection is crucial for successful treatment. Consider imaging studies (e.g., CT scan of the abdomen) to identify potential sources of infection, such as intra-abdominal abscesses or urinary tract infections. If a specific source is identified, targeted antimicrobial therapy can be considered.

3. De-escalation of Therapy: Once the source of infection is identified and culture results are available, the antibiotic regimen should be de-escalated to the narrowest spectrum of effective antibiotics. This approach can help minimize the risk of antibiotic-associated adverse effects and promote the growth of normal gut flora.

4. Monitoring for Adverse Effects: Closely monitor the patient for signs of antibiotic-associated adverse effects, such as Clostridium difficile infection, allergic reactions, or kidney dysfunction. Adjust the antibiotic regimen as needed based on the patient's clinical response and laboratory results.

Additional Considerations:

- **Antibiotic Susceptibility Testing:** Once culture results are available, perform susceptibility testing to guide optimal antibiotic therapy.
- **Infection Control Measures:** Implement strict infection control measures to prevent the spread of antibiotic-resistant organisms.
- **Supportive Care:** Continue to provide supportive care, including fluid resuscitation, vasopressor support (if needed), and pain management.

By following these recommendations, healthcare providers can optimize antibiotic therapy, minimize the risk of adverse effects, and improve patient outcomes.

Score:

Comments:

Q18 67-year-old male, known case of Hypertension, type 2 Diabetes, Chronic Obstructive Pulmonary Disease (COPD). Admitted to the ICU 48 hours ago following a severe case of community-acquired pneumonia resulting in respiratory failure. Currently intubated and mechanically ventilated, sedated , and on vasopressors to maintain blood pressure. Current Medications: Vasopressors (Norepinephrine), Sedation (Propofol), Antibiotics (Ceftriaxone and Azithromycin), Insulin sliding scale for glucose management, Vital Signs: Blood Pressure 100/60 mmHg on vasopressors, Heart Rate 85 bpm, Respiratory Rate 18 bpm (via ventilator). Lab Results: Elevated inflammatory markers, creatinine is stable at 1.1 mg/dL. What are the possible approaches regarding VTE (Venous Thromboembolism) prophylaxis?

This is for informational purposes only. For medical advice or diagnosis, consult a professional.

Given the patient's age, underlying medical conditions, and current critical care status, they are at high risk for venous thromboembolism (VTE). Therefore, aggressive VTE prophylaxis is recommended.

Here's a suggested approach:

Pharmacological Prophylaxis:

- **Low-Molecular-Weight Heparin (LMWH):** This is the preferred agent for VTE prophylaxis in critically ill patients. It has a favorable safety profile and is effective in reducing the risk of VTE.
- **Dose Adjustment:** The dose of LMWH should be adjusted based on the patient's renal function, as assessed by creatinine clearance.
- **Monitoring:** Closely monitor the patient for bleeding complications, especially if they are receiving additional medications that increase bleeding risk (e.g., anticoagulants, antiplatelet agents).

Mechanical Prophylaxis:

Intermittent Pneumatic Compression (IPC): This can be used as an additional measure to reduce the risk of VTE, especially in patients with contraindications to pharmacological prophylaxis or those at very high risk.

Important Considerations:

- **Risk Assessment:** Continuously assess the patient's risk of VTE and bleeding.

- **Individualized Approach:** The optimal approach to VTE prophylaxis should be tailored to the individual patient's clinical condition and risk factors.
- **Close Monitoring:** Closely monitor the patient for signs of bleeding or thrombosis.
- **Consult with a Specialist:** If there are concerns about the optimal approach to VTE prophylaxis, consult with a hematologist or critical care specialist.

Additional Considerations:

- **Underlying Medical Conditions:** The patient's underlying medical conditions, such as diabetes and COPD, can increase the risk of VTE.
- **Immobility:** Prolonged immobilization in the ICU increases the risk of VTE.
- **Inflammation:** Systemic inflammation associated with sepsis can further increase the risk of VTE.

By implementing effective VTE prophylaxis strategies, healthcare providers can significantly reduce the risk of VTE in critically ill patients and improve patient outcomes.

Score:

Comments:

END OF QUESTIONS – AI (2)-Platform
